# Epidemiological, clinical, and diagnostic characteristics of a large-scale upsurge of dengue in the rohingya refugee camps and host communities in Cox’s Bazar, Bangladesh, 2021 to 2024: A retrospective study

**DOI:** 10.1371/journal.pntd.0014198

**Published:** 2026-10-23

**Authors:** Charls Erik Halder, Md Abeed Hasan, Emmanuel Roba Soma, James Charles Okello, Md Mostafizur Rahman, Partha Pratim Das, U. Maung Prue, Dickson Wafula Barasa, Md Atiquzzaman, Md Sobuz Hosen, Saddam Hossain Shagar, Elizabeth Yie Chuen Chong, Debashish Paul, S. M. Niaz Mowla, Mohammadul Hoque, Abu Toha MHR Bhuiyan, Md Farhad Hussain

**Affiliations:** 1 Migration Health Division (MHD), International Organization for Migration (IOM), Cox’s Bazar, Bangladesh; 2 Migration Health Research Unit, Regional Office for Asia and the Pacific, International Organization for Migration (IOM), Bangkok, Thailand; 3 World Health Organization, Cox’s Bazar, Bangladesh; 4 Civil Surgeon Office, Cox’s Bazar, Bangladesh; 5 Refugee Relief & Repatriation Commissioner Office, Cox’s Bazar, Bangladesh; 6 Cox’s Bazar Medical College, Cox’s Bazar, Bangladesh; NIAID Integrated Research Facility, UNITED STATES OF AMERICA

## Abstract

**Background:**

Dengue emerged as a new public health threat in the Rohingya refugee camps in Cox’s Bazar, Bangladesh, in 2021 and expanded into large-scale upsurges in subsequent years. Evidence on dengue epidemiology and clinical presentation in protracted refugee settings remains limited, despite the need for stronger outbreak preparedness and case management in these contexts.

**Objectives:**

To describe the epidemiological, clinical, and diagnostic characteristics of the dengue upsurge among Rohingya refugees and surrounding host communities in Cox’s Bazar, Bangladesh, and to identify predictors of inpatient admission and diagnostic positivity patterns.

**Methods:**

This retrospective observational study used anonymized surveillance data from the International Organization for Migration (IOM) dengue patient database. Rapid diagnostic test (RDT)–confirmed dengue cases identified across 36 IOM-supported health facilities in Ukhiya and Teknaf between 1 October 2021 and 31 December 2024 were included. Demographic, epidemiological, clinical, and laboratory variables were summarized using descriptive statistics. Weekly case counts were aggregated by epidemiological week and calendar year, and epidemic growth and decay phases were modelled using phase-specific Poisson regression. Multivariable logistic regression was used to identify factors associated with inpatient admission, while separate models examined associations between delay in presentation and RDT positivity patterns, adjusting for age and sex.

**Results:**

A total of 35,581 RDT-confirmed dengue cases were reported, of which 90.2% occurred among Rohingya refugees. The median age was 17 years (IQR 7–30), and 46.0% of cases were among children aged 0–14 years. Annual caseload increased from 1,011 in 2021–11,752 in 2022, 10,669 in 2023 and 12,149 in 2024, with seasonal peaks during the monsoon period and progressively later peaks and longer epidemic tails over time. Poisson models showed decreasing growth rates across years (r = 0.449 in 2021 to r = 0.091 in 2024) with increasing doubling times, while decay rates remained broadly comparable (halving time ~4.4–6.0 weeks). Overall, 8.0% of cases required inpatient admission, 1.3% were referred, and four deaths were reported (case fatality <0.1%). In multivariable analysis, inpatient admission was associated with older age (≥60 vs 0–14: aOR 2.48), delayed presentation (aOR 1.06 per day), refugee status (aOR 1.43), fever ≥38°C at presentation (aOR 1.13), presence of any WHO warning sign (aOR 28.87), and chronic co-morbidity (aOR 6.63). Males had lower odds of admission than females (aOR 0.87).

**Conclusion:**

Sustained dengue preparedness is required in Cox’s Bazar, including strengthened surveillance, community-based early referral, targeted monitoring of high-risk groups, environmental vector control, and phase-appropriate use of NS1 and IgM/IgG diagnostics to reduce missed diagnoses and prevent progression to severe disease. These findings highlight the need for a policy shift from episodic outbreak response toward sustained dengue preparedness in humanitarian settings, including strengthened surveillance systems, integrated diagnostic strategies, community-based early referral, and coordinated vector control interventions.

## Author summary

Dengue has emerged as an important public health threat in the Rohingya refugee camps and surrounding host communities in Cox’s Bazar, Bangladesh. However, evidence describing dengue epidemiology and clinical presentation in refugee settings remains limited. We analyzed routinely collected surveillance data from 35,581 rapid diagnostic test-confirmed dengue cases attending 36 IOM-supported health facilities between October 2021 and December 2024. Dengue cases increased substantially after the initial outbreak in 2021, with seasonal peaks occurring during and after the monsoon period. Children represented a large proportion of reported cases, while older age, delayed presentation, chronic co-morbidities, and clinical warning signs were associated with inpatient admission. Diagnostic positivity also varied according to the timing of presentation, reflecting the expected transition from early NS1 antigen detection to later antibody detection. These findings demonstrate the growing operational burden of dengue in this prolonged humanitarian setting. Strengthening surveillance, early referral, diagnostic capacity, vector control, and preparedness may help humanitarian health systems identify cases earlier and respond more effectively to future dengue outbreaks.

## Introduction

Rohingya refugees have experienced multiple instances of forced displacement into Bangladesh as a result of conflict and persecution since 1978, with the largest displacement of Rohingya people from Myanmar to Bangladesh occurring in August 2017 [1,2]. As of 31st December 2025, approximately 1,148,571 Rohingya refugees were residing in 33 overcrowded camps in Ukhiya and Teknaf Upazilas of Cox’s Bazar [3,4]. The Rohingya refugees in Cox’s Bazar continue to face challenging living conditions characterized by overcrowded camps, limited access to essential resources and services, and increased vulnerability to natural and man-made disasters, like floods and landslides related to heavy monsoons rainfall, fire incidents and cyclones [1]. The crowded living arrangements with fragile shelters, inadequate water, sanitation, and hygiene (WASH) facilities and practices, coupled with intense monsoons rainfall in both the refugee camps and neighboring host communities, elevate their vulnerability to infectious diseases, frequently leading to disease outbreaks [1]. Since 2017, these areas have witnessed various episodes of infectious disease outbreaks or upsurges, including diphtheria, measles, COVID-19, and Acute Watery Diarrhea (AWD)/cholera [1,5]. Dengue appeared as a new threat for the Rohingya refugees from October 2021 which expanded as massive outbreaks in 2022 and 2023 [6–8].

Dengue is a viral infection caused by the Dengue virus (DENV), transmitted to humans through the bite of infected mosquitoes [9]. Despite DENV having four serotypes (DENV-1, DENV-2, DENV-3, DENV-4), infection with one serotype provides long-term immunity to only that serotype but not the others, with sequential infections raising the risk of severe dengue [10]. A patient with dengue can manifest with a spectrum of symptoms, ranging from mild to severe ones [9,10]. Common symptoms reported for dengue include high fever (40°C/104°F), severe headache, retro-orbital pain (pain behind the eyes), muscle and joint pains, nausea, vomiting, swollen glands, and rash [9,11]. Severe dengue symptoms often manifest after the fever has subsided and may include severe abdominal pain, persistent vomiting, rapid breathing, bleeding gums or nose, fatigue, restlessness, blood in vomit or stool, extreme thirst, pale and cold skin, and feeling weak [9,10]. Epidemiological and clinical characteristics of dengue have been described in several studies conducted over the past two decades, particularly from India, Myanmar, Thailand, and Malaysia [12–15]. However, most of the studies described the characteristics of dengue in urban setting or whole country setting [12,16]. Even the studies in Bangladesh also described the characteristics either mainly from the urban setting or based on the data from the whole country [16–18].

A few studies have been conducted on zoonotic diseases and applied research among displacement populations such as in internally displaced populations (IDP) camps in Sri Lanka and other global settings, yet a significant literature gap on the epidemiological and clinical characteristics of dengue still exists, especially within the Rohingya humanitarian context. This understanding is critical for projecting the nature of upcoming outbreaks and ensuring proper prevention, preparedness, and response strategies [1,7]. Previous studies in the Rohingya camps have also highlighted important gaps in preventive practices, community engagement, and risk communication during infectious disease responses, which remain highly relevant for dengue preparedness and response in this setting [5,19]. Recent studies from Bangladesh and other South and Southeast Asian settings have described variation in the clinical spectrum of dengue, including gastrointestinal and other non-classical manifestations, highlighting the importance of considering dengue among patients with diverse clinical presentations [15,20,21].

The Rohingya refugee camps in Bangladesh constitute a distinctive environment, hosting more than one million refugees living in improvised and overcrowded conditions marked by limited waste disposal, poor drainage systems, and makeshift shelters constructed from bamboo and tarpaulins [1]. An investigation into epidemiological factors is imperative for anticipating the likelihood, magnitude, duration, and peak timing of dengue outbreaks, facilitating the timely implementation of preventive measures and the establishment of preparedness and response plans [10,22]. Furthermore, an examination of the epidemiological and clinical features of dengue within this displaced setting is essential for formulating outbreak preparedness and response plans, strengthening case management measures while ensuring prompt implementation of risk communication and community engagement initiatives, and the forecasting and strategic prepositioning of medical commodities and supplies [10,23].

The objective of this study was to describe the epidemiological, clinical and diagnostic characteristics of large-scale dengue upsurge in the Rohingya refugee camps as well as in the surrounding host communities in Cox’s Bazar, Bangladesh. To be more specific, this study was designed to characterize transmission dynamics, clinical presentations, severity of the disease, pattern of presentation in health facilities, and diagnostic profiles of dengue cases in this protracted humanitarian setting. This study aimed to contribute to the evidence relevant to dengue outbreak preparedness, surveillance, and response strategies in displaced populations’ settings.

## Methodology

### Ethical statement

This study was conducted under a pre-approved protocol for secondary analysis of outbreak-related health and programme data. Ethical approval was obtained from the Ethical Review Board of Cox’s Bazar Medical College Hospital (CoxMC/2023/016). Administrative permission to use outbreak-response data was granted by the Office of the Civil Surgeon and the Refugee Relief and Repatriation Commissioner (RRRC). Internal approval was also obtained from the International Organization for Migration (IOM) Migration Health Research Unit for the study and retrospective use of programme data. Epidemiological and clinical case data were extracted from routine outbreak surveillance and clinical reporting databases used during the dengue response. These data were collected as part of public health outbreak response and health service delivery activities between 1 October 2021 and 31 December 2024 and subsequently used for research purposes under the approved secondary-use protocol. Anonymized surveillance database was accessed for research purposes between 01 August 2025 and 15 September 2025. All individual-level data were de-identified and anonymized prior to analysis, and only aggregate findings are reported. At no point did the investigators have access to direct or indirect patient identifiers. Given the retrospective nature of the study and the exclusive use of anonymized data, individual informed consent was not required.

### Study design

This was a retrospective observational study that utilized de-identified and anonymized data from the International Organization for Migration’s (IOM) dengue patient database in Cox’s Bazar, Bangladesh.

### Study setting and population

The study was implemented in Rohingya refugee camps in Ukhiya and Teknaf in Cox’s Bazar. The analytic study population comprised healthcare-attending patients with rapid diagnostic test (RDT)-confirmed dengue who received care at 36 IOM-supported health facilities in Ukhiya and Teknaf between 1 October 2021 and 31 December 2024. The dataset therefore represents facility-detected dengue cases rather than all dengue infections occurring in the refugee and surrounding host populations. Dengue was first detected in this displacement setting in 2021 and subsequently resulted in recurrent large outbreaks, prompting a substantial health response across the Rohingya camps.

### Data source

#### Dengue data collection and case definition.

De-identified and anonymized demographic, epidemiological, and clinical data pertaining to RDT-confirmed dengue cases of all ages among Rohingya refugees and surrounding host-community patients attending IOM-supported facilities were retrieved from the IOM dengue patient database for the period 1 October 2021–31 December 2024. All dengue cases were screened and identified across 36 IOM supported facilities in Ukhiya and Teknaf. The following case definitions were used for screening and diagnosis of the patients as per the national guideline for management of dengue syndrome in Bangladesh [23].

- Suspected dengue: Acute febrile illness with or without non-specific signs and symptoms.
- Confirmed dengue: Case patients reported as positive for NS1 antigen or IgM by rapid diagnostic tests

The analytic database contained RDT-confirmed cases only. Patients were considered RDT-confirmed when either NS1 antigen or IgM/IgG testing was positive. Testing was selected according to the timing of illness and clinical assessment, with NS1 antigen testing primarily used during the earlier phase of illness and IgM/IgG testing used during later presentation. The analytic dataset did not contain clinically suspected patients with negative RDT results; therefore, the number and proportion of suspected but RDT-negative dengue cases could not be evaluated retrospectively. Because the analytic database retained RDT-confirmed cases only, the total number of clinically suspected patients screened or tested, and the overall test-positivity proportion could not be reconstructed retrospectively. The health facilities collected daily data for demographic (e.g., age, sex, camp location), epidemiological clinical (e.g., symptoms, severity, complications, hospitalization, hospital stay, and referral), and laboratory findings (e.g., RDT result) of the cases using standard Case Report Form and Inpatient Admission Notification Form (in case of inpatient admission). All data were reported electronically using Kobo Toolbox and synchronized at the central database. Data was verified and cleaned centrally on a daily basis, then exported to a Microsoft Excel spreadsheet for further analysis.

#### Clinical management.

Out of 36 IOM-supported health facilities, 22 were health posts that operated only during daytime (8.00 AM to 4.00 PM), while 12 were 24/7 primary healthcare centers (PHCCs) with inpatient capacity, and 2 were integrated infectious disease treatment centers (IDTC) dedicated for management and admission of moderate to severe cases. The management of dengue cases followed the Bangladesh national treatment guidelines, which classified cases into groups A, B, and C corresponding to mild, moderate, and severe categories [23]. This classification aligns with the WHO’s revised classification (2009) for dengue hemorrhagic fever (DHF), which categorized dengue into three groups (A-B-C) to streamline case management based on disease severity (Fig 1). Group A patients were managed at all three levels of health facilities. IDTCs admitted Group B patients as well as patients with social factors (e.g., living far from the facility, living alone). IDTCs admitted patients referred from health posts or PHCCs (if the bed capacity is saturated). Group C patients were referred to District Sadar Hospital for initial stabilization. A dispatch and referral unit monitored real-time bed capacity across the health facilities, coordinated referrals, and dispatched ambulances. In addition, a network of Community Health Workers (CHWs) conducted daily follow-up of dengue cases managed in the outpatient departments (OPD) of the health facilities and monitored patients for warning signs using a standardized dengue community follow-up checklist.

**Fig 1:**
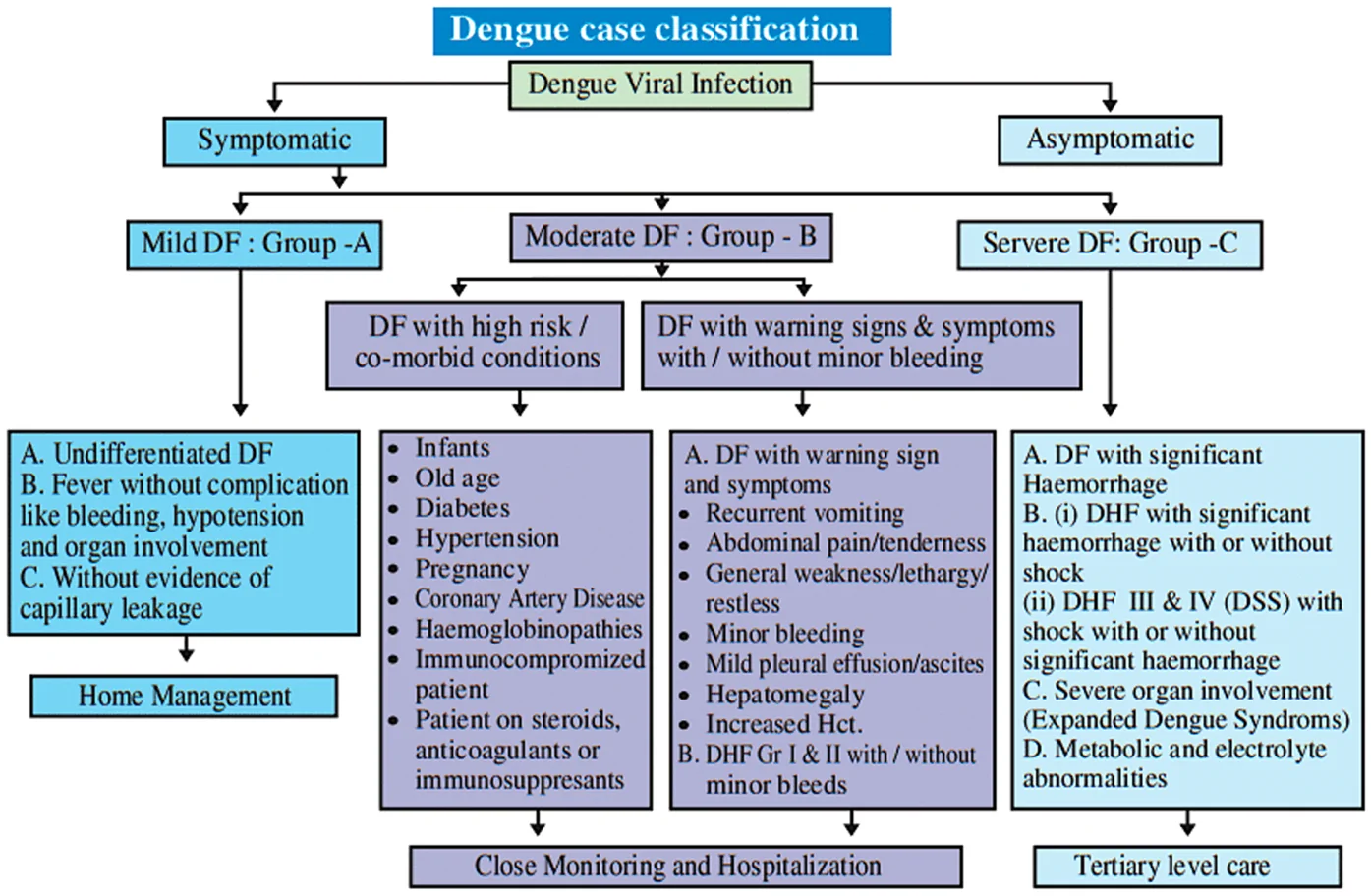
Dengue case classification used in clinical management (Groups A–C) [23].

#### Diagnostics.

NS1 antigen and IgM/IgG RDT kits were used across all facilities to diagnose dengue. Venous blood samples were collected from the patients for the tests. The NS1 antigen RDT was administered for patients in the early or febrile phase, while the IgM/IgG antibody RDT was utilized if the patient presented in the late or convalescence phase. This approach aligned with findings from systematic reviews, demonstrating the high sensitivity of combining NS1 and IgM tests during the respective phases of dengue. In instances where differentiation between febrile and convalescent phases was challenging, or diagnostic uncertainty persisted with a single RDT result, both tests were conducted. Information on the RDT manufacturer, lot number, product-specific sensitivity and specificity, and lot-level quality-assurance procedures was not retained in the retrospective analytic database. Molecular confirmatory test results were also not available in the analytic dataset. These limitations were considered when interpreting RDT-confirmed cases, particularly IgM-only positive cases.

### Data analysis

Demographic, epidemiological, and clinical characteristics were summarized using descriptive statistics, including frequencies, percentages, means, medians, standard deviations, and interquartile ranges, as appropriate. Population denominators were derived from the estimated catchment populations of the IOM-supported health facilities in Ukhiya and Teknaf. Year-specific catchment population estimates were 594,676 in 2021, 632,634 in 2022, 658,993 in 2023, and 691,857 in 2024, comprising the refugee populations of 19 IOM catchment camps and the populations of seven selected host-community unions. Refugee population estimates were primarily derived from UNHCR population data, while host-community estimates were based on Bangladesh Bureau of Statistics population projections and adjusted to the IOM facility catchment areas where applicable. Annual crude dengue rates were calculated as the number of RDT-confirmed cases recorded in each year divided by the corresponding year-specific catchment population and expressed per 10,000 population. Demographic subgroup rates presented in Table 1 used the corresponding available subgroup catchment denominators. Crude rate ratios and corresponding p-values for demographic subgroup comparisons were estimated using Poisson regression, with the specified reference category for each comparison. Detailed derivation of the annual catchment population estimates is provided in S1 Table. These catchment denominators represent annual programmatic population estimates and may not fully capture intra-year changes due to migration, births, deaths, and population mobility; formal uncertainty intervals around the denominator estimates were not available. Demographic variables were age, sex, population type (refugee or host community) as well as the geographical location. Clinical characteristics included symptoms during presentation, vital signs, presence of dengue warning signs as per WHO, presence of co-morbidities including pregnancy status and non-communicable diseases. This also captured the delay from onset of symptoms to presentation at a health facility. Diagnostic variables were RDT results (NS1 antigen and IgM). The standardized dengue case report form used for routine data collection is provided in S1 File.

**Table 1:** Distribution of dengue cases and crude reported case rates by demographic characteristics across 2021 to 2024 (N = 35,581).

Variable	Category	Dengue Count	Percentage (%)	Population	Crude reported case rate per 10,000	Crude rate ratio	P value
Overall	Total	35,581	100.00%	691,857	514.3	1.000	–
	Mean age (years)		20.02 ± 16.8				
	Median age (years)		17				
Sex	Male	19,349	54.40%	338,672	571.1	1.000	Ref
	Female	16,232	45.60%	353,185	459.5	0.80	<0.001
Age group	0 – 14 years	16,371	46.00%	359,766	455.0	1.000	Ref
	15 –29 years	10,141	28.5%	166,046	610.7	1.34	<0.001
	30 – 59 years	7,655	21.5%	138,371	553.2	1.22	<0.001
	≥60 years	1,414	4.0%	27,674	510.9	1.12	<0.001
Upazila	Ukhiya	30,259	85.00%	530,560	570.4	1.000	Ref
	Teknaf	5,275	14.80%	161,297	327.0	0.57	<0.001
Community	Host	3,504	9.80%	113,659	308.3	1.000	Ref
	Refugee	32,077	90.20%	578,198	554.6	1.80	<0.001

Population denominators presented in this table correspond to the 2024 IOM catchment population and are used as reference denominators for descriptive comparison of the cumulative 2021–2024 case series. These measures should not be interpreted as annual incidence rates or cumulative attack rates. Annual crude reported dengue rates were calculated separately using the corresponding year-specific catchment population denominators.

Weekly dengue case counts were aggregated by epidemiological week and calendar year according to the date of clinical examination. Annual epidemic curves were constructed for 2021–2024. For each calendar year, the peak week was defined as the epidemiological week with the highest observed weekly case count. The growth phase extended from the first epidemiological week included in the annual epidemic series through the peak week, and the decay phase extended from the week immediately following the peak through the final epidemiological week included in that year’s epidemic series. Phase-specific Poisson regression models were used to summarize weekly growth and decay patterns. Model estimates with 95% confidence intervals are presented in Table 3, and fitted trajectories are overlaid on observed weekly case counts in Fig 3. These models were intended primarily as descriptive summaries of epidemic trajectories rather than fully specified time-series models; potential overdispersion, temporal autocorrelation, and meteorological seasonality were not explicitly modelled and are therefore considered when interpreting the estimates.

Delay in presentation was analyzed as a continuous variable and also compared across RDT positivity patterns using non-parametric methods. The distribution of RDT positivity patterns by delay from symptom onset to presentation is illustrated in S1 Fig. Differences in delay across RDT categories were assessed using the Kruskal–Wallis test. Multivariable logistic regression models were used to examine the association between delay in presentation and RDT positivity patterns, with NS1 antigen-only positivity used as the reference category. Models were adjusted for age and sex, and results were reported as adjusted odds ratios (aORs) with 95% confidence intervals.

Data completeness was assessed before multivariable modelling. Variables included in the primary admission model were complete, whereas blood pressure measurements were substantially incomplete. Systolic and diastolic BP measurements were available for 18,864 of 35,581 patients (53.0%). Missingness was strongly age-dependent, with systolic BP recorded for only 7.2% of patients aged 0–14 years compared with 89.0% of those aged 15–29 years, 95.2% of those aged 30–59 years, and 96.6% of those aged ≥60 years. To avoid substantial age-dependent selection introduced by complete-case analysis, blood pressure variables were excluded from the primary multivariable model. A summary of variable completeness and missingness for the primary multivariable admission analysis is provided in S3 Table.

Inpatient admission was treated as a clinical and programme-management outcome rather than a direct proxy for biological disease severity. In addition to clinical findings, admission decisions could be influenced by social and operational factors, including distance from the health facility, living circumstances, referral pathways, and available inpatient capacity.

To examine factors associated with inpatient admission, multivariable logistic regression was performed using the complete analytic cohort. Covariates were selected a priori based on clinical relevance, WHO dengue severity criteria, and potential epidemiological confounding. The primary model included age group, sex, delay from symptom onset to presentation, population type, calendar year, fever ≥38°C at presentation, presence of any WHO warning sign, pregnancy, chronic co-morbidity, and previous dengue infection. Blood pressure variables were excluded from the primary model because of substantial and strongly age-dependent missingness. Adjusted odds ratios (aORs) with 95% confidence intervals were reported. Model discrimination was evaluated using the area under the receiver operating characteristic curve (AUC), calibration was assessed using decile-based calibration and the Hosmer-Lemeshow goodness-of-fit test, and multicollinearity was assessed using variance inflation factors. The primary model included 35,581 observations and 2,863 admission events, corresponding to approximately 204 admission events per estimated model parameter. All analyses were conducted using R statistical software (version 4.4.2; R Core Team, 2024).

### Research governance

This research aligned with the global strategy and priorities of IOM in relation to ensuring access to healthcare to save lives and protect the displaced populations. All existing literature, scientific evidence and ethical considerations were taken into account while designing the research protocol. Existing public health experts of IOM in the Migration Health Division, Cox’s Bazar took part in the research. While the first researcher (as mentioned under the research team) led the technical aspects of the research including protocol development, ethical consideration, training, data collection and analysis, and reporting, the second researcher led the research coordination, monitoring, budget allocation, and risk management. Other researchers took part in data collection, cleaning, analysis and review. All research team members (CV attached) possessed the relevant professional qualifications to contribute to the research study. Both first and second researchers did regular monitoring of timely implementation and quality of every step of the research. Training was organized for research team members on ethics, data collection, data protection, analysis and presentation.

### Limitations

This study has several limitations. First, the analysis included only healthcare-attending patients with RDT-confirmed dengue identified through 36 IOM-supported health facilities and therefore does not represent all dengue infections occurring in the refugee and surrounding host populations. Mild or asymptomatic infections, patients who did not seek care, and clinically suspected cases with negative RDT results were not captured, introducing potential selection and surveillance bias. Second, molecular confirmation, serotyping, and genotyping were unavailable, and information on RDT manufacturer, lot-specific performance, sensitivity, specificity, and quality-assurance procedures was not retained in the analytic database. Misclassification is therefore possible, particularly among IgM-only positive cases. The absence of serotyping and genotyping also prevented assessment of whether circulating viral strains contributed to differences in clinical severity, as reported in previous studies from Bangladesh [24]. Third, the retrospective use of routine programme data resulted in incomplete measurement of some variables, particularly blood pressure, and several potentially important factors, including socioeconomic status, distance to health facilities, healthcare-access differences, and referral pathways, were not fully captured. The admission model also did not account for potential clustering of patients within health facilities or camps; consequently, standard errors may be underestimated if outcomes were correlated within these units. Admission should consequently be interpreted as a clinical and programme-management outcome rather than a direct measure of disease severity. Fourth, deaths occurring outside participating facilities may not have been completely captured, and the very low observed case fatality rate may therefore underestimate overall dengue mortality. Fifth, the findings are derived from IOM-supported facility catchment populations and may not be generalizable to all of Cox’s Bazar or to populations using other healthcare providers. Finally, the absence of entomological, seroprevalence, meteorological, and viral serotype data limits interpretation of the observed temporal patterns and prevents firm conclusions regarding endemicity or the mechanisms underlying sustained transmission. The phase-specific count models also did not explicitly account for temporal autocorrelation or seasonal meteorological covariates, and the estimated growth and decay parameters should therefore be interpreted primarily as descriptive temporal measures. Overdispersion was also not explicitly modelled; therefore, uncertainty from the simple Poisson models may be underestimated if the variance in weekly counts exceeded the Poisson assumption. In addition, demographic subgroup rates in Table 1 use the 2024 catchment population as a reference denominator for the cumulative 2021–2024 case series and should therefore be interpreted as descriptive burden measures rather than annual incidence or cumulative attack rates. Because dengue transmission was first detected in October 2021, comparisons between 2021 and the complete calendar years of 2022–2024 should also be interpreted cautiously.

## Result

### Demographics

A total of 35,581 rapid diagnostic test confirmed dengue positive cases were reported from the selected health facilities in the Rohingya refugee camps, of which 90.2% of the cases were among Rohingya refugees and rest were among local Bangladeshi communities. Using year-specific catchment population denominators, the annual crude reported dengue rate was 17.0 per 10,000 population in 2021, 185.8 per 10,000 in 2022, 161.9 per 10,000 in 2023, and 175.6 per 10,000 in 2024. (Table 1).

The median age of the cases was 17 years (IQR 7 – 30 years). Nearly half of all reported dengue cases occurred among children aged 0–14 years. The majority of cases were documented in Ukhiya Upazila (85.0%), followed by Teknaf (14.8%). A higher proportion of cases was observed among males compared with females (54.4% vs. 45.6%) (Table 2).

**Table 2:** Demographic characteristics of RDT-confirmed dengue cases at presentation across 2021 to 2024 (N = 35,581).

Characteristic	Category	n (%) or value
Overall caseload	Total cases	35,581 (100.0)
Sex	Female	16,232 (45.6)
	Male	19,349 (54.4)
Age (years)	Mean (SD)	20.2 (16.8)
	Median (IQR)	17 (7–30)
	Range	0.003–117
Age group distribution	0–14 years	16,371 (46.0)
	15–29 years	10,141 (28.5)
	30–59 years	7,655 (21.5)
	≥60 years	1,414 (4.0)
Population type	Host community	3,504 (9.8)
	Refugee	32,077 (90.2)
Geographic distribution (Upazila)	Ukhiya	30,259 (85.0)
	Teknaf	5,275 (14.8)
	Ramu	25 (0.1)
	Cox’s Bazar Sadar	22 (0.1)

### Epidemiological characteristics

Fig 2 illustrates the weekly trends in dengue cases by year, highlighting variations in epidemic timing, with progressively later peaks and extended epidemic tails over successive years. The initial outbreak was reported in October 2021, corresponding to the lowest overall case count (1,011), which increased in subsequent years (11,752 in 2022; 10,669 in 2023; and 12,149 in 2024). In all years, case numbers remained relatively low during the early epidemiological weeks, followed by a marked seasonal increase during the Monsoon period, with peaks occurring between weeks 25 and 38, varying by year. Notably, 2022 saw an early surge with a pronounced peak around weeks 25–26, while 2023 displayed a delayed and less acute peak near week 30. In contrast, 2024 experienced a prolonged epidemic characterized by a later peak during weeks 34–36 and sustained elevated incidence prior to decline.

**Fig 2:**
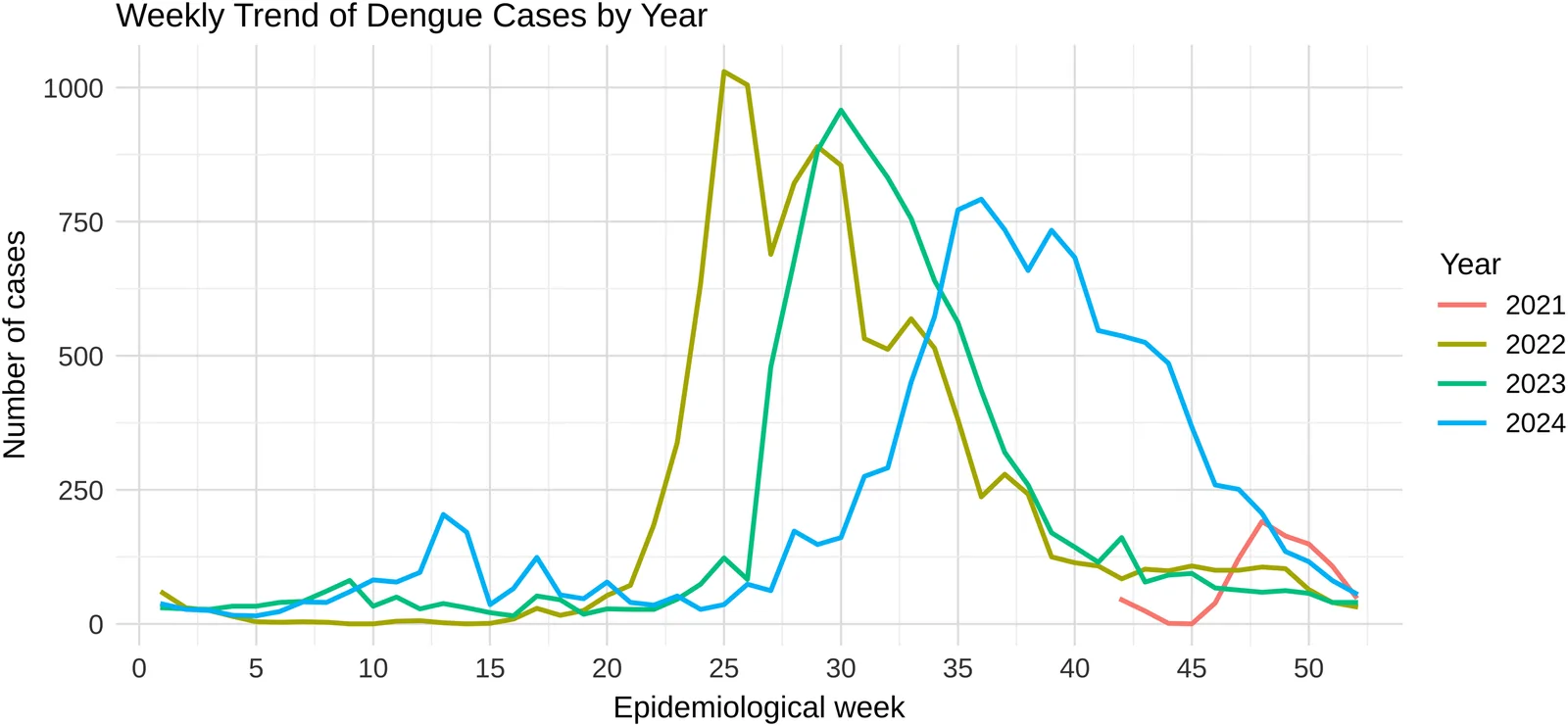
Weekly dengue cases by epidemiological week and year (2021–2024).

A phase-specific Poisson regression analysis demonstrates clear growth and decay phases in dengue transmission across all years (2021–2024) with notable variations in epidemic expansion and decline (Table 3, Fig 3). In October 2021, transmission began with a rapid surge and sharp decline, characterized by the fastest growth rate (r = 0.449; 95% CI: 0.394–0.506) and the shortest doubling time of 1.54 weeks. This was followed by a significant decline (r = −0.285; 95% CI: −0.364 to −0.208), with a halving time of 2.43 weeks. As surveillance began in October 2021, the 2021 growth and decay estimates should be interpreted cautiously, as the shorter observation window may have influenced the observed epidemic trajectory. From 2022 to 2024, epidemic growth became progressively slower, with reduced growth rates (r = 0.299 in 2022; 0.149 in 2023; 0.091 in 2024) and corresponding increase in doubling times, lengthened from 2.32 to 7.61 weeks. In contrast, post-peak decay rates remained generally comparable across years (ranging from −0.116 to −0.159), with halving times between 4.37 and 5.97 weeks, indicating more prolonged epidemic persistence despite attenuated transmission intensity.

**Table 3:** Phase-specific weekly epidemic growth and decay parameters from Poisson regression (2021–2024).

Year	Phase	r (per week)	95% CI	Doubling/ Halving time (weeks)
2021	Decay	−0.285	−0.364 to −0.208	2.43 (halving)
2021	Growth	0.449	0.394 to 0.506	1.54 (doubling)
2022	Decay	−0.116	−0.119 to −0.113	5.97 (halving)
2022	Growth	0.299	0.288 to 0.311	2.32 (doubling)
2023	Decay	−0.159	−0.164 to −0.153	4.37 (halving)
2023	Growth	0.149	0.144 to 0.154	4.67 (doubling)
2024	Decay	−0.131	−0.137 to −0.125	5.30 (halving)
2024	Growth	0.091	0.088 to 0.094	7.61 (doubling)

**Fig 3:**
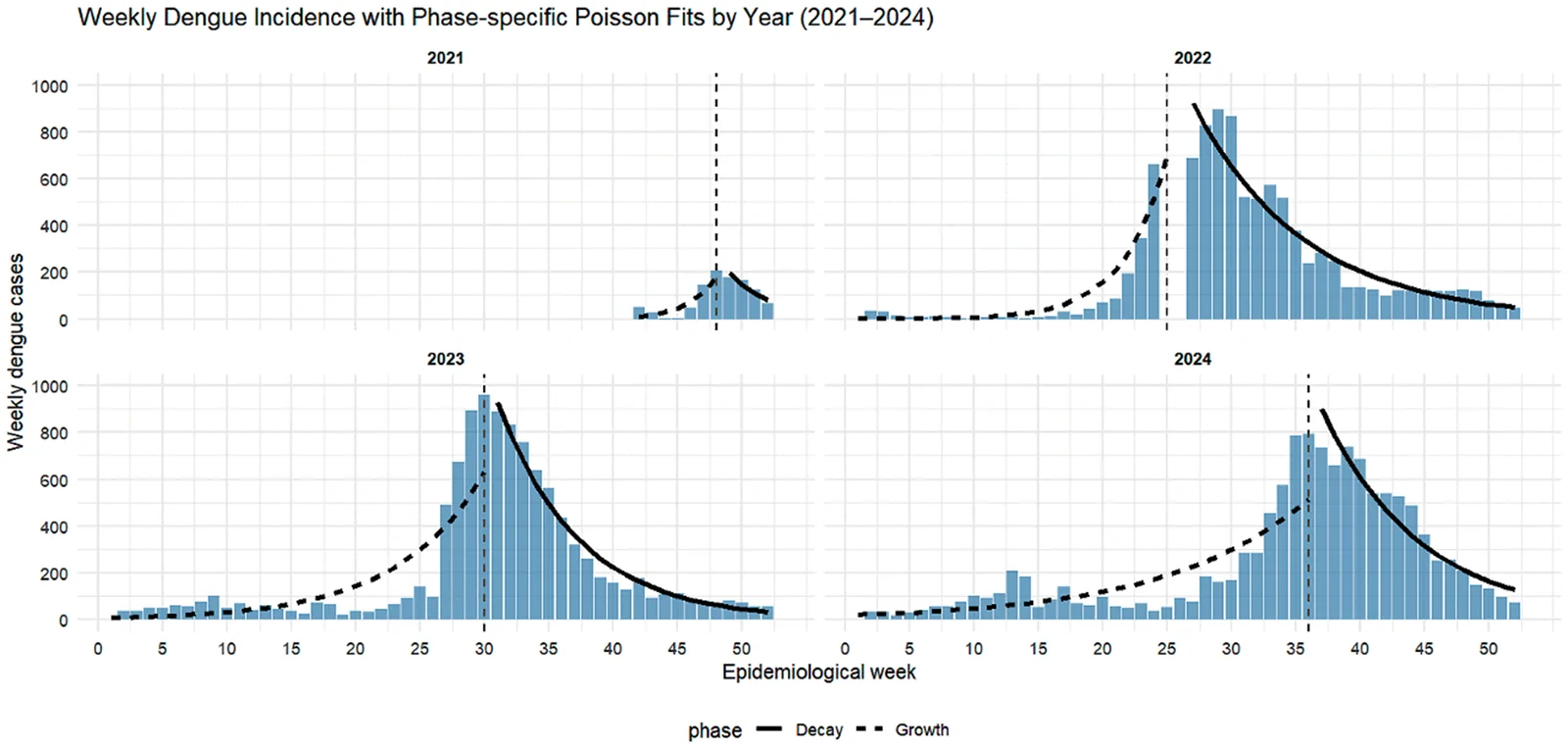
Weekly dengue case counts with phase-specific Poisson regression fits, Cox’s Bazar, 2021–2024. Bars represent observed weekly RDT-confirmed dengue cases. Fitted curves represent phase-specific Poisson regression estimates for the growth and decay periods. The vertical dashed line indicates the observed annual peak week separating the growth and decay phases.

### Clinical characteristics

Of the 35,581 cases reviewed, 8% (2,863 cases) were admitted for inpatient care. The majority, 90.6% (32,223 cases), were managed on an outpatient basis, while only 1.3% required referral to a tertiary facility. There were four reported deaths, resulting in a case fatality rate of less than 0.1%. (Table 4)

**Table 4:** Clinical profile and management outcomes among dengue cases across 2021-2024 (N = 35,581).

A. Management and clinical outcome	
**Characteristic**	**n (%)**
**Admitted**	2,863 (8.0)
**Not admitted**	32,718 (92.0)
OPD treatment	32,223 (90.6)
Referred	466 (1.3)
Discharged on same day	21 (0.1)
Absconded	4 (<0.1)
Died at home	3 (<0.1)
Died in facility	1 (<0.1)
**B. Disease severity (WHO classification)**	
**Severity category**	**n (%)**
Group A: Mild dengue	31,836 (89.5)
Group B: Dengue with warning signs	3,587 (10.1)
Group C: Severe dengue	158 (0.4)
**C. Clinical diagnosis**	
**Clinical diagnosis**	**n (%)**
Asymptomatic dengue	111 (0.3)
Dengue fever	35,369 (99.4)
Dengue hemorrhagic fever	35 (0.1)
Dengue shock syndrome	60 (0.2)
Dengue with multiorgan failure	6 (<0.1)

Fig 4 illustrates the distribution of symptoms reported by the patients during their first presentation. Other than documented fever, dengue cases are most frequently presented with headache (58.7%) and malaise (55.1%). One third of cases presented with cough (30.8%). A substantial proportion of patients presented with gastrointestinal symptoms including nausea (18.8%), anorexia (18.4%), vomiting (14.0%) and abdominal pain (5.6%). Musculoskeletal symptoms included joint pain (16.3%) and body ache (3.8%). Retro-orbital pain was reported among 7.5% of the participants. Severe manifestations were very rarely reported, which include bleeding manifestation (0.3%), rash (0.1%), hypotension (0.1%) delirium (1.7%), respiratory distress (0.1%) and convulsion (<0.1%).

**Fig 4:**
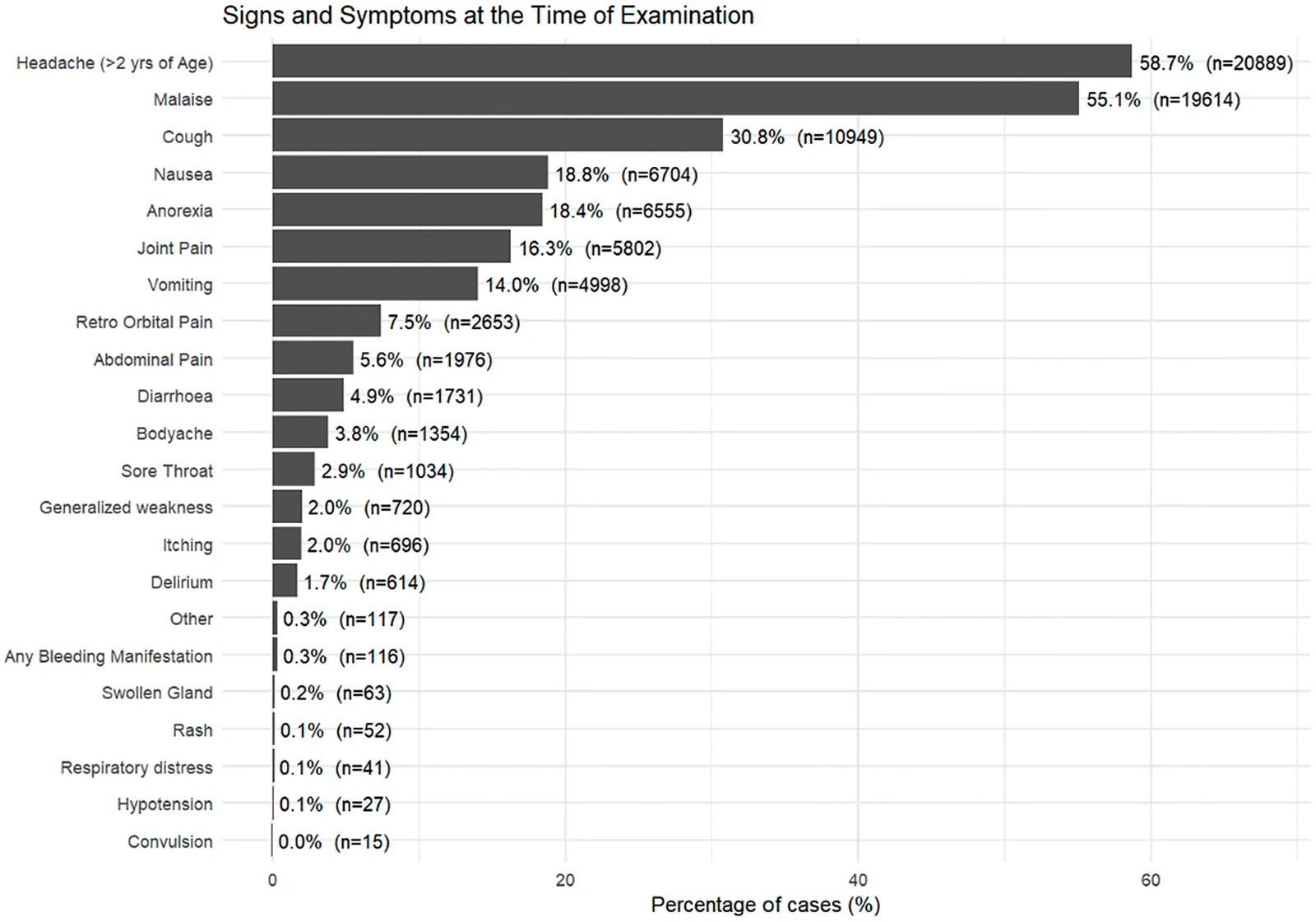
Frequency of symptoms reported at first clinical presentation among RDT-confirmed dengue cases (N = 35,581).

While over half of the patients had a recorded fever (≥38 °C), other vital signs of the majority of the patients stayed within normal ranges (Table 5). Low systolic BP (<90 mmHg) was observed in 2.8% of cases, and low diastolic BP (<60 mmHg) appeared in 6.4%. Furthermore, 4.6% of patients exhibited a prolonged capillary refill time (>2 seconds).

**Table 5:** Vital signs at presentation among dengue patients.

Vital sign	Valid records (n)	Mean	Median	Mode	Observed range	Abnormal value (%)
Body temperature (°C)	35,581	37.6	38	37	36–42	Fever ≥38 °C: 50.2%
Systolic BP (mmHg)	18,864	108.3	109	100	40–196	<90 mmHg: 2.8%
Diastolic BP (mmHg)	18,864	69.1	70	70	30–140	<60 mmHg: 6.4%
Capillary refill time (sec)	35,581	1.55	2	1	1–4	>2 sec: 4.6%

WHO warning signs were observed in only a small proportion of patients as illustrated in Table 6. The most frequently reported warning signs included abdominal pain (3.6%), lethargy or restlessness (3.3%), and persistent vomiting (2.2%). Less commonly noted warning signs were present in fewer than 0.2% of cases, comprising of mucosal bleeding, clinical fluid accumulation, laboratory evidence of rising haematocrit, and liver enlargement exceeding 2 cm. Co-existing morbidities include infancy (2.9%) and old age (2.3%), pregnancy (1.5%) and chronic non-communicable diseases (Table 6).

**Table 6:** WHO warning signs and co-existing morbidities at presentation across 2021 to 2024 (N = 35,581).

WHO warning sign	n (%)
Abdominal pain	1,294 (3.6%)
Lethargy/ restlessness	1,181 (3.3%)
Persistent vomiting	769 (2.2%)
Mucosal bleeding	58 (0.2%)
Clinical fluid accumulation	18 (0.1%)
Laboratory: increase in haematocrit	13 (<0.1%)
Liver enlargement >2 cm	11 (<0.1%)
Co-existing morbidities	
Extreme age	
Infancy	1,031 (2.9%)
Old age	801 (2.3%)
Pregnancy	523 (1.5%)
Chronic non-communicable diseases	
Hypertension	200 (0.6%)
Diabetes mellitus	162 (0.5%)
Bronchial asthma	51 (0.1%)
Heart disease	31 (0.1%)
Stroke	13 (<0.1%)
Chronic haemolytic anaemia	10 (<0.1%)
Obesity	9 (<0.1%)
Renal failure	4 (<0.1%)

WHO warning signs were not mutually exclusive; individual patients could have more than one warning sign recorded.

Multivariable logistic regression identified several demographic and clinical factors associated with inpatient admission (Table 7). The likelihood of inpatient admission grew with age: individuals aged 15–29 had an adjusted odds ratio (aOR) of 1.68 (95% CI: 1.50–1.88), those aged 30–59 had an aOR of 1.98 (95% CI: 1.76–2.22), and people aged 60 or older had an aOR of 2.48 (95% CI: 2.04-3.01) when compared to children aged 0–14. Male patients had lower odds of admission than females (aOR 0.87; 95% CI 0.79–0.95). Delay in presentation from symptom onset was associated with increased odds of inpatient admission (aOR 1.06; 95% CI 1.04–1.08). Refugees had significantly higher odds of admission compared with host community patients (aOR 1.43; 95% CI 1.22–1.69). Clinically, the presence of any WHO dengue warning sign was strongly associated with hospital admission (aOR 28.87; 95% CI 26.19–31.82). Patients with any chronic co-morbidity had over sixfold increased risk of inpatient admission compared with those without comorbid conditions (aOR 6.63; 95% CI 5.15–8.54). Fever ≥38°C at presentation was modestly associated with higher odds of inpatient admission (aOR 1.13, 95% CI 1.03–1.24), whereas pregnancy (aOR 1.04, 95% CI 0.72–1.48) and previous dengue infection (aOR 1.11, 95% CI 0.80–1.56) were not independently associated with admission. Admission odds were found higher in 2021 (aOR 3.97, 95% CI 3.26–4.83) and modestly lower in 2024 (aOR 0.87, 95% CI 0.77–0.97) in comparison to 2022.

**Table 7:** Multivariable logistic regression predictors of inpatient admission among dengue patients cumulatively from 2021-2024.

Predictor	Adjusted OR	95% CI	p-value
Age group			
15–29 vs 0–14	1.68	1.50–1.88	<0.001
30–59 vs 0–14	1.98	1.76–2.22	<0.001
≥ 60 vs 0–14	2.48	2.04–3.01	<0.001
Male vs female	0.87	0.79–0.95	0.002
Delay in presentation, per day	1.06	1.04–1.08	<0.001
Refugee vs host community	1.43	1.22–1.69	<0.001
Calendar year			
2021 vs 2022	3.97	3.26–4.83	<0.001
2023 vs 2022	1.10	0.98–1.23	0.117
2024 vs 2022	0.87	0.77–0.97	0.014
Fever ≥38°C	1.13	1.03–1.24	0.013
Any WHO warning sign	28.87	26.19–31.82	<0.001
Pregnancy	1.04	0.72–1.48	0.844
Chronic co-morbidity	6.63	5.15–8.54	<0.001
Previous dengue	1.11	0.80–1.56	0.530

A sensitivity analysis excluding WHO warning signs demonstrated that the direction and magnitude of the main associations with age, sex, delayed presentation, refugee status, and chronic co-morbidity remained broadly consistent, supporting the robustness of the primary model findings (S2 Table). The multicollinearity assessment demonstrated that the adjusted GVIF values were uniformly low (1.006–1.041), indicating no evidence of problematic multicollinearity among the clinically related predictors.

The final multivariable model included all 35,581 patients, with 2,863 admission events and 204.5 events per estimated parameter, indicating ample information relative to model complexity. The model provided significantly better fit than the intercept-only model (likelihood-ratio χ^2^ = 5,452, df = 14, p < 0.001; AIC = 14,496.38) and showed good discrimination for inpatient admission (AUC 0.828, 95% CI 0.818–0.837). Although the Hosmer–Lemeshow test was statistically significant (χ^2^ = 42.92, df = 8, p < 0.001), this should be interpreted in the context of the large sample size; predicted and observed admission proportions were broadly similar across most risk deciles, indicating reasonable but not perfect calibration (S2 Fig).

### Exposure and behavioral characteristics

Table 8 summarizes the exposure history and behavioral characteristics of the dengue cases. Around 80% of the patients reported a history of increased mosquito bites in past three weeks and more than 60% reported the presence of stagnant water in their close surroundings. However, majority of the patients reported using at least one personal protective measure against mosquito bites. A small proportion (1.5%) of the patients reported a previous dengue episode.

**Table 8:** Exposure history and preventive factors among dengue cases (N = 35,581).

Variable	Category	n (%)
Previous dengue infection	No	35,036 (98.5)
	Yes	545 (1.5)
Travel within 10 days before onset	No	35,272 (99.1)
	Yes	309 (0.9)
Increased mosquito bites in past 3 weeks	No	7,223 (20.3)
	Yes	28,358 (79.7)
Stagnant water in close surroundings	No	13,627 (38.3)
	Yes	21,954 (61.7)
Family member with similar symptoms (past 10 days)	No	32,155 (90.4)
	Yes	3,426 (9.6)
Use of protective measures	No	4,273 (12.0)
	Yes	31,308 (88.0)

### Diagnostic characteristics

Among the total pool of dengue patients, NS1 antigen alone was positive in 62.1%, IgM alone was positive in 33.6%, and both NS1 antigen and IgM/IgG were positive in 4.3% of the cases. Table 9 indicates a correlation between the delay from symptom onset to clinical presentation and RDT positivity patterns. Patients exhibiting NS1 antigen positivity exclusively reported the shortest interval before seeking medical care (mean 2.4 days, median 2 days). Those positive for both NS1 antigen and IgM/IgG antibodies experienced an intermediate delay (mean 4.1 days, median 4 days), while the longest delay was noted among patients positive solely for IgM/IgG antibodies (mean 5.0 days, median 5 days). After adjusting for age and sex, each additional day of delay from symptom onset to presentation was independently associated with higher odds of being IgM-only positive (aOR 2.43, 95% CI 2.38–2.47; p < 0.001).

**Table 9:** Delay in presentation by RDT positivity pattern (N = 35,581).

RDT positivity pattern	n (%)	Mean delay (days)	Median delay (days)	Kruskal–Wallis test
NS1 antigen only positive	22,082 (62.1)	2.4	2	χ² = 12,319, df = 2, p < 0.001
Both NS1 antigen and IgM/IgG positive	1,544 (4.3)	4.1	4	
IgM/IgG only positive	11,955 (33.6)	5	5	
**Total**	**35,581 (100)**	—	—	

## Discussion

This study describes a large, multi-year dengue outbreak among Rohingya refugees and surrounding host communities in Cox’s Bazar, providing epidemiological and clinical evidence from a humanitarian setting where published dengue data remain limited [1]. This retrospective analysis confirms the exceptionally high burden of dengue among the Rohingya refugees in Cox’s Bazar with a caseload of more than 35,000 from 2021 to 2024. Extremely crowded living conditions with fragile and crowded shelters may contribute to the high transmission in the refugee setting as this provides abundance of human hosts for mosquitoes [10]. Poor sewerage systems, stagnation of water near the households and poor waste management system may further contribute to the transmission by increasing the habitats for mosquitoes [6,25]. This is further evidenced by finding of this study, as more than 60% of participants reported the presence of stagnant water in their close surroundings (Table 8). These findings indicate that dengue prevention strategies in the Rohingya camps must prioritize environmental vector control and improved water, sanitation, and waste management systems through stronger coordination between health, WASH, and camp management sectors.

Our study identified a significant seasonal increase in dengue cases, with peaks observed between weeks 25 and 38, corresponding to the late- and post-monsoon period, consistent with previously reported dengue seasonality in Bangladesh [16] This pattern suggests that water stagnation following rainfall contributes to mosquito breeding and subsequent dengue transmission [10]. The analysis indicates that dengue emerged as an acute outbreak in 2021, followed by larger-scale outbreaks with progressively slower growth rates in subsequent years. The reasons for the progressively slower epidemic growth observed across successive years cannot be determined from these data and may reflect changes in population susceptibility, surveillance and healthcare-seeking patterns, intervention coverage, vector dynamics, or other unmeasured factors. Apart from the seasonal peaks, dengue cases were also reported during lower-transmission periods across successive years. The progressively shifting peak periods may also reflect environmental or seasonal influences; however, rainfall, humidity, and temperature were not directly evaluated in this analysis [10]. Therefore, there is critical need for shifting priorities from short-term outbreak response towards the sustained dengue preparedness, surveillance, and vector control. From a policy perspective, the recurrence of dengue transmission across successive years requires integrating dengue preparedness into routine health system planning, including year-round surveillance, vector monitoring, and contingency resource allocation within humanitarian health programmes. These recurrent transmission patterns suggest that dengue control strategies in the Rohingya camps should shift from short-term outbreak response toward sustained preparedness measures, including year-round surveillance, proactive vector control, and long-term programme planning.

We have found that the males accounted for a higher proportion of cases than females. This is consistent with the findings in other settings like the countries in South and South-east Asia, including Dhaka, Bangladesh [15]. While the researchers yet could not conclude the underlying cause of the male predominance of dengue, in our setting, it could be related with the fact that men culturally spend more time engaged in outside activities and occupations that increase their likelihood to get exposure [16].

The study reported the spectrum of presenting symptoms for patients with dengue. While this study reported a significant proportion of common dengue symptoms, atypical symptoms such as cough, abdominal pain and diarrhea were also observed across all four years [11,21]. Previous studies in urban settings in Bangladesh suggest that Bangladeshi healthcare workers have documented an increasing trend in gastrointestinal symptoms and a decreasing trend in classical hemorrhagic manifestations (like skin petechiae or melena) and joint pain in dengue cases [15], emphasizing the need for clinicians to maintain a high index of suspicion for dengue even in patients presenting with non-classical symptoms, particularly in high-transmission and resource-limited settings such as refugee camps. For clinical practice and programme planning, this highlights the need to strengthen clinician awareness across healthcare facilities and ensure that up-to-date diagnostic triaging protocols for dengue are available.

Overall, only four deaths were recorded among the 35,581 RDT-confirmed cases in the surveillance dataset, corresponding to a reported case fatality of 0.011%. This low recorded fatality should be interpreted cautiously because the surveillance system primarily captured patients attending IOM-supported facilities and may not have identified all deaths occurring in the community or outside the participating health facilities. The relatively young case population, healthcare access, circulating serotypes, early diagnosis and referral practices, and incomplete mortality ascertainment may all have contributed to the observed rate. Early detection, timely surveillance, and prompt clinical consultation play a critical role in reducing dengue severity and mortality by enabling early case management, rapid identification of outbreaks, and timely referral of high-risk patients [10]. These findings suggest that maintaining decentralized diagnostic capacity, standardized clinical protocols, and community-based follow-up systems should remain central components of dengue control policies in humanitarian health settings.

Only 1.5% of cases had a documented history of previous dengue infection; however, this should not be interpreted as evidence that the remaining cases represented primary infections, because previous asymptomatic or undocumented infections could not be excluded. Evidence suggests that subsequent dengue infections increase the risk of severe disease compared with primary infections due to mechanisms such as antibody-dependent enhancement (ADE), particularly when reinfection is caused by a different dengue virus serotype [9]. Considering high population density, favorable climate for vector proliferation, inadequate vector control measures in the Rohingya refugee camps, there is a heightened likelihood of repeated exposure and secondary infections in the future, which may consequently increase the risk of severe dengue cases if preventive and surveillance measures are not strengthened [16]. Policy planning should therefore prioritize sustained vector control, strengthened surveillance systems, and preparedness for potential increases in severe dengue associated with secondary infections.

Multivariable regression analysis indicated that inpatient admission was associated with age, delayed presentation, population type, WHO warning signs, chronic co-morbidity, and fever at presentation, whereas pregnancy and previous dengue infection were not independently associated with admission. The higher adjusted odds of admission among refugee patients should be interpreted cautiously. Admission may also have been influenced by differences in healthcare access, facility distribution, referral pathways, distance to inpatient services, living circumstances, and socioeconomic factors that were not fully captured in the surveillance database. Residual confounding therefore cannot be excluded.

Delayed presentation exposes patients to the critical phase of dengue, during which plasma leakage, hypotension, and organ dysfunction are more likely to occur, thereby increasing the need for inpatient admission; this relationship has been consistently reported in clinical and epidemiological studies from dengue-endemic, resource-limited settings [10,26,27]. Therefore, risk communication and community engagement should be strengthened for increasing health seeking behavior, community-based referrals and rapid triage to prevent delays in presentation. This is consistent with earlier studies from the Rohingya camps that documented challenges in community engagement, preventive practices, and trust-building during infectious disease responses, underscoring the importance of strengthening community-based communication strategies in dengue control efforts [5,19]. The strong association between WHO warning signs and inpatient admission should not be interpreted as an independent predictor of biological disease severity, because warning signs form part of the clinical criteria used to determine admission. The magnitude of this association therefore largely reflects clinical decision-making and application of dengue management guidelines. Strengthening community-based early detection and referral systems may therefore reduce delays in care-seeking and prevent progression to severe disease requiring hospitalization.

The study found that Calendar year was independently associated with inpatient admission. The higher admission odds in 2021 likely reflect the early outbreak period, when precautionary admission practices and lower admission thresholds may have been more common, rather than greater disease severity. By 2024, admission odds were modestly lower than in 2022, which may indicate evolving case-management practices and greater experience in managing dengue at outpatient level.

The study confirmed predominant NS1 antigen positivity among patients presenting early after symptom onset and IgM antibody positivity among those presenting later, with a brief overlapping period during which both markers were detectable. This pattern aligns with the pathophysiology of dengue infection, in which NS1 antigenemia is highest during the early febrile phase (typically within the first 3–5 days of illness) and declines as viremia resolves, while dengue-specific IgM antibodies become detectable from approximately day 4–5 onward and persist into the convalescent phase. Consequently, in humanitarian settings where delayed care-seeking is common, reliance on NS1-based testing alone would fail to identify a substantial proportion of dengue cases. For patients presenting later in the course of illness, IgM/IgG RDTs may complement NS1 testing and improve case detection. However, IgM-only positivity should be interpreted cautiously because dengue-specific IgM may persist beyond the acute phase, and molecular confirmation was not available in this study. This finding is consistent with current World Health Organization and Centers for Disease Control and Prevention guidance, which recommend serological testing for patients presenting beyond the early febrile phase of illness [10,28–30]. Ensuring the availability of both NS1 antigen and IgM/IgG rapid diagnostic tests across primary healthcare facilities is therefore essential for accurate case detection and appropriate clinical management in humanitarian settings.

The findings from this research provide several important policy insights. The recurrence of dengue outbreaks across successive years in Cox’s Bazar highlights the need to move from predominantly episodic emergency response toward sustained preparedness. Delayed care-seeking and diagnostic delays indicate the importance of strengthening surveillance, risk communication and community engagement, referral, and diagnostic services at primary health facilities. Furthermore, the environmental and exposure findings support stronger intersectoral coordination for vector control and environmental management. Overall, strengthening these measures is essential to reduce dengue transmission, promote earlier presentation to health facilities, and improve morbidity and mortality outcomes among dengue patients.

## Conclusion

The study documented the epidemiological, clinical and diagnostic characteristics of a large multi-year dengue outbreak among Rohingya refugees and surrounding host communities in Cox’s Bazar. Recurrent outbreaks and dengue case detection across successive years suggest the possibility of increasingly sustained transmission, although the available data do not establish endemicity. The combination of high population density, environmental suitability for vector breeding, and delayed care-seeking underscores the urgent need to shift from episodic outbreak response to a long-term dengue control strategy. This should include sustained vector control and environmental management, strengthened routine and syndromic surveillance, integrated use of NS1 and IgM/IgG diagnostics across all phases of illness, and continued investment in community-based risk communication, early referral, and triage systems. Proactive identification and close monitoring of high-risk groups — particularly older adults, patients with co-morbidities, and those presenting late — will be critical to preventing progression to severe disease. Strengthening these components within humanitarian health systems is essential to mitigating the future burden of dengue in protracted displacement settings such as Cox’s Bazar.

## Supporting information

S1 Table Derivation of annual catchment population denominators for IOM-supported health facilities in Ukhiya and Teknaf, Cox’s Bazar, 2021–2024. The table presents year-specific population estimates for the 19 refugee camps and seven host-community unions included in the IOM health-facility catchment area, together with the sources and assumptions used to derive the annual denominators.
(XLSX)

S2 Table Sensitivity analysis of predictors of inpatient admission excluding WHO warning signs. Multivariable logistic regression model examining factors associated with inpatient admission after excluding the WHO warning-sign variable from the model. Adjusted odds ratios (aORs), 95% confidence intervals (CIs), and p-values are presented for age group, sex, delay in presentation, population type, calendar year, fever ≥38°C, pregnancy, chronic co-morbidity, and previous dengue infection.
(DOCX)

S3 Table Completeness of variables considered in the primary multivariable admission analysis. Completeness and missingness of variables considered for inclusion in the primary multivariable logistic regression model. Blood pressure variables were excluded from the primary model because of substantial and strongly age-dependent missingness. No imputation was performed.
(DOCX)

S1 Fig RDT positivity patterns by delay from symptom onset to presentation. Distribution of NS1 antigen–only, IgM/IgG–only, and dual RDT positivity patterns according to delay in days from symptom onset to presentation among dengue cases.
(TIF)

S2 Fig Calibration of the primary multivariable logistic regression model for inpatient admission. Observed and predicted probabilities of inpatient admission across risk deciles for the primary multivariable logistic regression model.
(TIF)

S1 File Dengue case report form used for routine outbreak surveillance and clinical data collection. Standardized case reporting form capturing demographic, epidemiological, clinical, laboratory, and outcome variables for dengue patients across IOM-supported health facilities.
(DOCX)

S1 Checklist Human Participants Research Checklist. (DOCX)
