# Supplementary material for: Epidemiological, clinical, and diagnostic characteristics of a large-scale upsurge of dengue in the rohingya refugee camps and host communities in Cox’s Bazar, Bangladesh, 2021 to 2024: A retrospective study
Source: PLoS Negl Trop Dis. 2026 Oct 23;20(10):e0014198. doi: 10.1371/journal.pntd.0014198 (PMC13719038; doi:10.1371/journal.pntd.0014198)
Supplement: Supplementary file 1 [file pntd.0014198.s002.docx]

**S2 Table. Sensitivity analysis of predictors of inpatient admission excluding WHO warning signs.** Multivariable logistic regression model examining factors associated with inpatient admission after excluding the WHO warning-sign variable from the model. Adjusted odds ratios (aORs), 95% confidence intervals (CIs), and p-values are presented for age group, sex, delay in presentation, population type, calendar year, fever ≥38°C, pregnancy, chronic co-morbidity, and previous dengue infection.

| **Predictor** | **aOR** | **95% CI** | **p-value** |
| --- | --- | --- | --- |
| Age 15–29 vs 0–14 | 1.81 | 1.63–2.00 | <0.001 |
| Age 30–59 vs 0–14 | 2.11 | 1.90–2.33 | <0.001 |
| Age ≥60 vs 0–14 | 3.03 | 2.57–3.57 | <0.001 |
| Male vs female | 0.83 | 0.77–0.90 | <0.001 |
| Delay, per day | 1.05 | 1.04–1.07 | <0.001 |
| Refugee vs host | 1.53 | 1.32–1.76 | <0.001 |
| 2021 vs 2022 | 2.87 | 2.42–3.42 | <0.001 |
| 2023 vs 2022 | 1.11 | 1.01–1.23 | 0.037 |
| 2024 vs 2022 | 0.90 | 0.81–0.99 | 0.030 |
| Fever ≥38°C | 1.17 | 1.08–1.27 | <0.001 |
| Pregnancy | 0.87 | 0.64–1.19 | 0.390 |
| Chronic co-morbidity | 6.93 | 5.63–8.52 | <0.001 |
| Previous dengue | 1.37 | 1.04–1.81 | 0.027 |
