# Supplementary material for: Epidemiological, clinical, and diagnostic characteristics of a large-scale upsurge of dengue in the rohingya refugee camps and host communities in Cox’s Bazar, Bangladesh, 2021 to 2024: A retrospective study
Source: PLoS Negl Trop Dis. 2026 Oct 23;20(10):e0014198. doi: 10.1371/journal.pntd.0014198 (PMC13719038; doi:10.1371/journal.pntd.0014198)
Supplement: Supplementary file 2 [file pntd.0014198.s003.docx]

**S3 Table. Completeness of variables considered in the primary multivariable admission analysis.** Completeness and missingness of variables considered for inclusion in the primary multivariable logistic regression model. Blood pressure variables were excluded from the primary model because of substantial and strongly age-dependent missingness. No imputation was performed.

| **Variable** | **Total N** | **Available n** | **Missing n** | **Missing (%)** |
| --- | --- | --- | --- | --- |
| Inpatient admission | 35,581 | 35,581 | 0 | 0.0 |
| Age group | 35,581 | 35,581 | 0 | 0.0 |
| Sex | 35,581 | 35,581 | 0 | 0.0 |
| Delay from symptom onset to presentation | 35,581 | 35,581 | 0 | 0.0 |
| Population type | 35,581 | 35,581 | 0 | 0.0 |
| Calendar year | 35,581 | 35,581 | 0 | 0.0 |
| Fever ≥38°C | 35,581 | 35,581 | 0 | 0.0 |
| Any WHO warning sign | 35,581 | 35,581 | 0 | 0.0 |
| Pregnancy | 35,581 | 35,581 | 0 | 0.0 |
| Chronic co-morbidity | 35,581 | 35,581 | 0 | 0.0 |
| Previous dengue infection | 35,581 | 35,581 | 0 | 0.0 |
| Systolic blood pressure* | 35,581 | 18,864 | 16,717 | 47.0 |
| Diastolic blood pressure* | 35,581 | 18,864 | 16,717 | 47.0 |

**Footnote:**
Blood pressure variables were excluded from the primary multivariable model because of substantial missingness (47.0%), which was strongly age-dependent and could have introduced selection bias through complete-case analysis. No imputation was performed.
