# Supplementary material for: Epidemiological, clinical, and diagnostic characteristics of a large-scale upsurge of dengue in the rohingya refugee camps and host communities in Cox’s Bazar, Bangladesh, 2021 to 2024: A retrospective study
Source: PLoS Negl Trop Dis. 2026 Oct 23;20(10):e0014198. doi: 10.1371/journal.pntd.0014198 (PMC13719038; doi:10.1371/journal.pntd.0014198)
Supplement: Supplementary file 3 [file pntd.0014198.s006.docx]

**Dengue Case Report Form**

**Report date**

No date selected: ___________________________________________________

**Facility Details**

Facility Name: _____________________________________________________

Facility Code: ______________________________________________________

1. **Case Identification Details**

Case ID: _________________________________________________________

Name of the Patient: _______________________________________________

Guardian's Name: _________________________________________________

Phone Number: ___________________________________________________

Age:_______	Unit: Days/Month/Year

Sex: M/F

Nationality: Host/Refugee

2. **Demographic Details**

District: ____________________________________________________________

Upazilla: ___________________________________________________________

Union: _____________________________________________________________

Camp: _____________________________________________________________

Block: _____________________________________________________________

Subblock: __________________________________________________________

Household number: __________________________________________________

Majhee Name: ______________________________________________________

Majhee phone number: _______________________________________________

3. **Clinical Information**

Date of Examination: __________________________________________________

Date of Symptom Onset: _______________________________________________

Date of Fever Onset: : __________________________________________________

Temperature at the time of Examination (F) : ________________________________

**Symptoms at the time of Examination**

**Symptoms (check)**

- Cough
- Sore Throat
- Malaise
- Headache (>2 years of Age)
- Retro Orbital Pain
- Joint Pain
- Swollen Gland
- Itching
- Anorexia
- Abdominal Pain
- Nausea
- Vomiting
- Any Bleeding Manifestation
- Other

**Signs at the time of Examination**

Heart Rate (per minute): : ___________________________________________

Blood pressure (mm/Hg):

Systolic: : __________________________________________________

Diastolic: : __________________________________________________

Capillary Refill Time: ______________________________________________

Rash:	Yes/No

Conjunctivitis:	Yes/No

Haemorrhegic Sign:	Yes/No

Tourniquet Test:	Yes/No

Other Signs : __________________________________________________

4. **Warning Signs:**

**Warning signs:**

- Abdominal pain
- Persistent vomiting
- Clinical fluid accumulation
- Mucosal bleeding
- Lethargy/restlessness
- Liver enlargement >2cm
- Laboratory: increase in HCT
- Other (specify): __________________

**Coexisting morbidity:**

- Pregnancy
- Infancy
- Old age
- Diabetes mellitus

**Social circumstances:**

- Living alone
- Living far from hospital
- Other:

**Severe signs:**

- Severe plasma leakage
- Shock
- Fluid accumulation with respiratory distress
- Severe bleeding
- Severe organ impairment

**Information on Risk Factor**

Did the patient suffer from Dengue Fever Before? Yes/No

Did the patient travel to another places 10 days before the onset of fever? Yes/No

**Travel Details**

Place Visited:

Duration of visit: ____ days

**Exposure History**

Did you see increase mosquito bites in past 3 week?	Yes/No

Stagnant Water in Close surrounding	Yes/No

Any family member got similar symptoms in last 10 days	Yes/No

Did the patient use any protective measures?	Yes/No

**Hospital Admission**

Date of hospital admission

**Treatment received**

Did the patient require transfusion?	Yes/No

**Specimen Collection**

Was a specimen collected?	Yes/No

Specimen Types:	Blood/serum

Specimen Collection Date:

**Test Type:**	RDT/serology/other

**Test results:**

RDT for NS1 Ag:	Positive/Negative/Not done

RDT for IgG/IgM:	Positive/Negative/Not done

WBC count (if done):

Platelet count (if done):

Other test results (if done):

**Diagnosis and Outcome**

Clinical Diagnosis

- Asymptomatic dengue
- Dengue fever
- Dengue hemorrhagic fever
- Dengue shock syndrome
- Dengue with multiorgan d

Additional diagnosis:

Category:

- Group A: mild dengue
- Group B: moderate dengue/dengue with warning sign
- Group C: severe dengue

Outcome

- Discharged
- Referred to PHC
- Referred to tertiary facility
- Absconded
- Died in facility
- Died at home
- Recovered
- Other: specify

Date of Outcome

Name of CHW following up the case: ________________________________

**Follow-up outcome:**

**Follow-up date:**

**Follow-up outcome:**

- Recovered
- Developed warning sign/severity
- Died in facility
- Died at home
- Other: specify

**Referred by CHW?	Y/N**

**Referred within the same day of complication?	Y/N**

**Type of Complication Reported by CHW: __________________________________________**

**Warning/severe sign found during follow-up at health facility:**

**Warning signs:**

- Abdominal pain
- Persistent vomiting
- Clinical fluid accumulation
- Mucosal bleeding
- Lethargy/restlessness
- Liver enlargement >2cm
- Laboratory: increase in HCT
- Other (specify): __________________

**Severe signs:**

- Severe plasma leakage
- Shock
- Fluid accumulation with respiratory distress
- Severe bleeding
- Severe organ impairment
