# Supplementary material for: Epidemiological, clinical, and diagnostic characteristics of a large-scale upsurge of dengue in the rohingya refugee camps and host communities in Cox’s Bazar, Bangladesh, 2021 to 2024: A retrospective study
Source: PLoS Negl Trop Dis. 2026 Oct 23;20(10):e0014198. doi: 10.1371/journal.pntd.0014198 (PMC13719038; doi:10.1371/journal.pntd.0014198)
Supplement: Supplementary file 5 [file pntd.0014198.s008.docx]

**Comments for Manuscript Number PNTD-D-26-00603**

Major comments

1. Selection bias & case definition (Major concern)

Only RDT-confirmed cases were included.

This excludes:

Clinically suspected dengue

False negatives (especially late NS1 or early IgM windows)

Problem issues: Attack rates, Severity estimates, Diagnostic pattern interpretation

Suggested revision:

Explicitly discuss sensitivity/specificity limitations of NS1/IgM

Provide:

Testing algorithm flow (who gets tested?)

Proportion of suspected vs tested vs confirmed cases

Consider sensitivity analysis if data available

2. Denominator & attack Rate validity

Attack rates are reported (e.g., 514.3 per 10,000 person-years) but:

Population denominator (691,857) is unclear

Refugee population is dynamic (migration, births, mobility)

Suggested revision:

Clarify: Source and stability of population denominators; whether yearly denominators were adjusted;

Add uncertainty

3. Poisson regression for epidemic growth

Weekly counts modeled using simple Poisson regression

No adjustment for:

Overdispersion

Temporal autocorrelation

Seasonality (rainfall, temperature)

Suggested revision:

Test for overdispersion -consider negative binomial

If possible consider: Time-series models (e.g., GAM, ARIMA)

Clarify how epidemic start/end defined (subjective?)

5. Extremely low case fatality rate (<0.1%)

Only 4 deaths out of 35,581 cases reported

This is unusually low for large dengue outbreaks

Suggested revision:

Explicitly discuss:

1. Mortality surveillance completeness
2. Community deaths vs facility deaths
3. Avoid over-attributing success to intervention model

6. Confounding in regression models

But missing key confounders:

1. Socioeconomic status
2. Distance to facility
3. Camp-level clustering
4. Time (year/epidemic phase)

Suggested revision:

Include:

Year as covariate

Facility-level clustering (random effects if possible)

Clarify variable selection strategy beyond “a priori”

8. Generalizability

Data from IOM-supported facilities only (36 sites)

Not representative of: Entire Cox’s Bazar and Non-IOM healthcare access

Suggested revision:

Clearly state limited external validity

Avoid broad claims like “largest outbreak globally” unless justified

9. Inconsistency in age Distribution

Table 1 appears inconsistent (e.g., % vs counts mismatch for age groups)

Needs verification and correction

10. Clinical severity underestimation

Severe dengue only 0.4%

Warning signs ~10%

Likely due to: Early detection bias or RDT-only inclusion

12. No serotype / genotype data

Acknowledged limitation but: Critical for interpreting outbreak dynamics

13. Figure clarity

Figures (pages 14–16) are informative but: Lack confidence intervals and overlaid model fits not clearly explained

Minor comments

Grammar issues:

“its’ largest displacement” is incorrect possessive

Redundancy in conclusion (repeated policy statements)

Overuse of long sentences (reduce for readability)

Ensure consistent terminology:

“upsurge”, “outbreak”, “epidemic”

**This manuscript presents a valuable and large-scale analysis of dengue epidemiology in a highly vulnerable humanitarian setting. The dataset is impressive and addresses an important evidence gap. However, there are several critical methodological and interpretational issues that limit the robustness of the conclusions. In particular, concerns around selection bias due to RDT-confirmed case inclusion, unclear population denominators for attack rate estimation, lack of adjustment for overdispersion in Poisson models, and potential residual confounding in regression analyses need to be addressed. Additionally, the extremely low case fatality rate requires further clarification regarding surveillance completeness. The diagnostic analysis largely confirms expected biological patterns and should be reframed accordingly. I recommend major revision focusing on methodological transparency, cautious interpretation of findings, and strengthening of analytical approaches.**
