# Supplementary material for: Epidemiological, clinical, and diagnostic characteristics of a large-scale upsurge of dengue in the rohingya refugee camps and host communities in Cox’s Bazar, Bangladesh, 2021 to 2024: A retrospective study
Source: PLoS Negl Trop Dis. 2026 Oct 23;20(10):e0014198. doi: 10.1371/journal.pntd.0014198 (PMC13719038; doi:10.1371/journal.pntd.0014198)
Supplement: Supplementary file 6 [file pntd.0014198.s009.docx]

**Major Comments**

1. **Lines 141-143**

The study defines "confirmed dengue" as positivity by NS1 or IgM RDT alone.

**Concern:**

IgM positivity alone does not necessarily indicate acute dengue infection and may persist for weeks to months after infection. WHO generally considers laboratory confirmation through PCR, virus isolation, or seroconversion stronger evidence than IgM RDT alone.

**Recommendation:**

- Provide sensitivity and specificity of the RDTs used.
- Clarify whether quality assurance or confirmatory laboratory testing was performed.
- Discuss the possibility of misclassification, particularly among IgM-only positive cases.

**2. Attack Rate Calculations Need Further Explanation**

**Lines 262-264 and Table 1**

The cumulative attack rate is reported as 514.3 per 10,000 person-years.

**Concern:**

The denominator is unclear.

Questions:

- Were annual population estimates used?
- Were refugee population changes between 2021 and 2024 accounted for?
- How were host-community denominators estimated?

**Recommendation:**

Provide a detailed explanation of denominator derivation in Methods.

**3. Potential Selection Bias**

**Lines 128-132**

Cases were identified from 36 IOM-supported facilities.

**Concern:**

The manuscript repeatedly discusses dengue epidemiology in the entire refugee population, but surveillance only covers healthcare-seeking patients.

**Recommendation:**

Explicitly state that:

- Findings represent healthcare-attending dengue cases.
- Attack rates may underestimate community transmission.
- Mild/asymptomatic infections are likely missed.

**4. Admission Outcome Is Influenced by Programmatic Decisions**

**Lines 150-164; 356-367**

The primary regression outcome is inpatient admission.

**Concern:**

Admission was partly determined by:

- social factors,
- distance from facility,
- bed availability.

Therefore admission may not accurately reflect disease severity.

**Recommendation:**

Discuss how operational factors may have influenced admission decisions and potentially biased regression estimates.

**5. Logistic Regression Model Requires More Details**

**Lines 206-215**

Important model diagnostics are missing.

**Please report:**

- Number of observations included in final model
- Missing data handling
- Events-per-variable ratio
- Variable selection strategy
- AUC confidence interval
- Hosmer-Lemeshow p-value

Without these details, model validity cannot be assessed.

**6. Warning Sign Variable Causes Circularity**

**Lines 362-363**

Presence of WHO warning signs is associated with admission (aOR 26.6).

**Concern:**

This is expected because warning signs are part of admission criteria.

Thus the result is somewhat tautological.

**Recommendation:**

Either:

- remove this variable from interpretation, or
- acknowledge that this association reflects clinical decision-making rather than independent disease risk.

**7. Strong Conclusions About Endemicity Are Not Fully Supported**

**Lines 423-425**

Authors state that dengue is "taking endemic form."

**Concern:**

No entomological data, seroprevalence data, or multi-year incidence modelling is presented.

**Recommendation:**

Use more cautious language:

"The findings suggest the possibility of transition toward sustained transmission."

rather than claiming endemicity.

**8. Refugee vs Host Community Comparison Needs Adjustment**

**Lines 361-362**

Refugees had higher odds of admission.

**Concern:**

Potential confounding by:

- healthcare access,
- referral pathways,
- facility distribution,
- socioeconomic factors.

**Recommendation:**

Discuss residual confounding and avoid causal interpretation.

**9. Diagnostic Timing Analysis Is Predictable**

**Lines 394-400**

The finding that delayed presentation is associated with IgM positivity is biologically expected.

**Concern:**

This section contributes limited novel knowledge.

**Recommendation:**

Consider shortening this analysis or moving detailed results to supplementary material.

**10. Missing Spatial Analysis**

Given the size of the dataset (35,000+ cases across 33 camps), absence of spatial analysis is a missed opportunity.

**Recommendation:**

Include:

- camp-level incidence map,
- hotspot analysis,
- yearly spatial distribution.

This would substantially strengthen the manuscript for PLOS NTD.

**Minor Comments**

**Introduction**

**Line 62**

"its' largest displacement"

Should be:

"its largest displacement"

**Lines 87-89**

Sentence structure is awkward.

Consider revision for clarity.

**Lines 98-100**

Authors claim atypical dengue presentation.

Provide stronger evidence and references specific to Bangladesh or South Asia.

**Methods**

**Line 149**

"excel spreadsheet"

Capitalize appropriately:

"Microsoft Excel"

**Lines 191-196**

Poisson regression assumptions should be justified.

Were overdispersion diagnostics assessed?

If overdispersion exists, negative binomial regression may be more appropriate.

**Lines 197-198**

Please explain how epidemic onset was defined.

**Results**

**Table 1 (Page 13)**

Several values appear inconsistent.

For example:

Age group 0-14 years:

- count reported as 1,414
- percentage reported as ~52%

This appears incorrect.

Table should be carefully checked.

**Table 1**

Percentages shown as "~52%", "~24%", "~20%", "~4%" are unusual.

Provide exact percentages.

**Line 269**

"The median age of the cases were"

Should be:

"The median age of the cases was"

**Line 314**

"8% (2,863 cases) necessitated inpatient admission"

Prefer:

"8.0% (2,863/35,581) were admitted"

**Table 5**

Only 18,864 BP measurements available.

Please explain why nearly half the cohort lacks BP data.

**Table 6**

Clarify whether warning signs were mutually exclusive or could overlap.

**Discussion**

**Lines 406-407**

Claim:

"largest outbreak of dengue recorded in the refugee or displacement setting globally"

This requires stronger evidence or should be softened.

**Lines 451-455**

The low CFR is attributed to the IOM response model.

This is speculative.

Alternative explanations include:

- younger population,
- healthcare access,
- circulating serotypes,
- under-ascertainment of deaths.

Please temper this interpretation.

**Additional Analyses That Would Strengthen the Paper**

If data are available, I strongly recommend:

1. **Year-specific admission models**
   - assess whether predictors changed over time.
2. **Camp-level hotspot mapping**
   - highly relevant for PLOS NTD.
3. **Age-specific epidemic curves**
   - children vs adults.
4. **Monthly positivity trends by diagnostic method**
   - NS1 vs IgM.
5. **Sensitivity analysis excluding IgM-only cases**
   - very important given the case definition.

**Final Recommendation**

**Major Revision**

The dataset is highly valuable and likely publishable in PLOS NTD, but the manuscript requires substantial strengthening of methodological transparency, validation of attack-rate estimates, clarification of admission models, correction of table inconsistencies, and more cautious interpretation of findings before it is suitable for publication.
