# Supplementary material for: Epidemiological, clinical, and diagnostic characteristics of a large-scale upsurge of dengue in the rohingya refugee camps and host communities in Cox’s Bazar, Bangladesh, 2021 to 2024: A retrospective study
Source: PLoS Negl Trop Dis. 2026 Oct 23;20(10):e0014198. doi: 10.1371/journal.pntd.0014198 (PMC13719038; doi:10.1371/journal.pntd.0014198)
Supplement: Supplementary file 7 [file pntd.0014198.s011.docx]

**Response to Editor and Reviewers**

**Manuscript ID:** PNTD-D-26-00603

**Title: *Epidemiological, Clinical, and Diagnostic Characteristics of a Large-Scale Upsurge of Dengue in the Rohingya Refugee Camps and Host Communities in Cox’s Bazar, Bangladesh, 2021 to 2024: A Retrospective Study***

Dear Editor and Reviewers,

We sincerely thank the Editor and Reviewers for their careful evaluation of our manuscript and for the constructive comments and recommendations. We have revised the manuscript extensively in response to these comments. The revisions have substantially strengthened the methodological transparency, statistical analysis, interpretation of findings, reporting of missing data, denominator calculations, model diagnostics, discussion of potential bias and confounding, and presentation of the supporting information.

All changes are shown in the tracked-changes version of the manuscript. A clean revised manuscript has also been provided. Our point-by-point responses are presented below.

**Additional Editor Comments**

**Comment 1**

Please provide an Author Summary of 150–200 words between the Abstract and Introduction.

**Response:**

Thank you. An Author Summary has been added between the Abstract and Introduction. It summarizes the main findings and public health relevance of the study in accessible language for both scientific and non-scientific readers.

**Change made:**

Author Summary added after the Abstract and before the Introduction.

**Comment 2**

Please remove supplementary tables from the manuscript file and upload them separately as Supporting Information. Please ensure that each Supporting Information file has a legend listed after the references.

**Response:**

Thank you. All supporting information items have now been organized separately and their legends are listed after the references in the manuscript.

The Supporting Information now includes:

- S1 Table: Derivation of annual catchment population denominators
- S2 Table: Sensitivity analysis excluding WHO warning signs
- S3 Table: Completeness of variables considered in the primary multivariable admission analysis
- S1 Figure: RDT positivity patterns by delay from symptom onset
- S2 Figure: Calibration of the primary multivariable admission model
- S1 File: Dengue case report form

**Change made:**

Supporting Information legends are provided after the references, and the corresponding files will be uploaded separately as Supporting Information.

**Comment 3**

Please double-check the amended Competing Interest statement.

**Response:**

We have reviewed the statement and confirm that it is correct.

**Change made:**

The manuscript states: “The authors declare no competing interests.”

**Reviewer 1**

**Major Comment 1. Selection bias and case definition**

**Comment:**

Only RDT-confirmed cases were included, excluding clinically suspected dengue and false-negative cases. The reviewer requested clarification of the testing algorithm, the proportion of suspected/tested/confirmed cases, and discussion of RDT sensitivity and specificity limitations.

**Response:**

We agree and have substantially clarified the case definition and selection process. The revised manuscript now explicitly states that the analytic population consisted of healthcare-attending, RDT-confirmed dengue cases identified at IOM-supported facilities and does not represent all dengue infections occurring in the community.

We also clarified that the analytic database contained only RDT-confirmed cases and did not retain clinically suspected RDT-negative cases. Therefore, the total number of suspected individuals screened or tested and the overall test-positivity proportion could not be reconstructed retrospectively.

We further expanded the diagnostic methods and limitations to note that information on the RDT manufacturer, lot number, product-specific sensitivity and specificity, and lot-level quality-assurance procedures was not retained in the analytic dataset. Molecular confirmation was also unavailable. The potential for misclassification, particularly among IgM-only positive cases, is now explicitly acknowledged.

**Change made:**

Revised Study Setting and Population, Data Source, Diagnostics, and Limitations sections.

**Major Comment 2. Denominator and attack-rate validity**

**Comment:**

The population denominator was unclear, the refugee population is dynamic, and it was unclear whether annual denominators were adjusted.

**Response:**

We agree and have revised the denominator methodology substantially. The revised manuscript now reports year-specific IOM catchment population estimates for each study year:

• 2021: 594,676

• 2022: 632,634

• 2023: 658,993

• 2024: 691,857

**Change made:**

Expanded Data Analysis section, revised Table 1 footnote, added S1 Table, and expanded Limitations.

Annual crude reported dengue rates were calculated using the corresponding year-specific catchment population. We also clarified that refugee estimates were primarily derived from UNHCR data, while host-community estimates were based on Bangladesh Bureau of Statistics projections and adjusted to the IOM facility catchment areas where applicable.

We further acknowledge that these are annual programmatic population estimates and may not fully capture intra-year population changes resulting from migration, births, deaths, and mobility. Formal uncertainty intervals for the population estimates were not available.

For Table 1, we now explicitly state that the 2024 catchment population is used only as a reference denominator for descriptive comparison of the cumulative 2021–2024 case series and should not be interpreted as annual incidence or cumulative attack rate.

**Major Comment 3. Poisson regression for epidemic growth**

**Comment:**

Simple Poisson regression did not account for overdispersion, temporal autocorrelation, or seasonality. The reviewer suggested testing for overdispersion, considering negative-binomial or time-series models, and clarifying epidemic onset/end definitions.

**Response:**

We appreciate this important comment. We clarified the definition of the annual peak week and the corresponding growth and decay phases and reframed the phase-specific Poisson models as descriptive summaries rather than fully specified time-series models. We did not add negative-binomial, GAM, or ARIMA models in the present revision. Accordingly, we now explicitly acknowledge that overdispersion, temporal autocorrelation, and meteorological seasonality were not modelled and that uncertainty from the Poisson models may therefore be underestimated. We also strengthened the Discussion by noting that the observed late- and post-monsoon seasonal pattern is consistent with previously reported dengue seasonality in Bangladesh.

**Change made:**

Revised Data Analysis and Limitations sections, and expanded Figure 3 legend.

**Major Comment 4. Extremely low case-fatality rate**

**Comment:**

Only four deaths were reported. The reviewer requested discussion of mortality surveillance completeness, community deaths, and avoidance of over-attribution to the intervention model.

**Response:**

We agree. The discussion has been revised to avoid attributing the low reported case-fatality rate to the IOM response model. We now explicitly state that deaths occurring outside participating facilities may not have been completely captured and that the observed CFR may therefore underestimate overall dengue mortality.

We also discuss alternative explanations, including the relatively young case population, healthcare access, circulating serotypes, early diagnosis and referral practices, and incomplete mortality ascertainment.

**Change made:**

Expanded Limitations and revised Discussion of mortality findings.

**Major Comment 5. Confounding in regression models**

**Comment:**

The reviewer identified potential residual confounding by socioeconomic status, distance to facility, camp-level clustering, and time/year, and recommended inclusion of year and facility-level clustering where possible.

**Response:**

We agree. Calendar year has now been included as an a priori covariate in the primary multivariable admission model.

The revised model includes age, sex, delay in presentation, population type, calendar year, fever ≥38°C, WHO warning signs, pregnancy, chronic co-morbidity, and previous dengue infection.

We also expanded the Methods to explain that covariates were selected a priori based on clinical relevance, WHO dengue severity criteria, and potential epidemiological confounding.

Facility- and camp-level random effects were not included in the final model. We therefore explicitly acknowledge in the Limitations that the model did not account for potential clustering within health facilities or camps and that standard errors may consequently be underestimated if outcomes were correlated within these units.

We also acknowledge that socioeconomic status, distance to facilities, healthcare access, referral pathways, and related factors were not fully captured.

**Change made:**

Revised Data Analysis, Table 7, Results, Discussion, and Limitations.

**Major Comment 6. Generalizability**

**Comment:**

Data came from IOM-supported facilities and may not represent all of Cox's Bazar.

**Response:**

We agree. The revised manuscript now explicitly states that the analytic population represents healthcare-attending, facility-detected dengue cases from IOM-supported facilities rather than all dengue infections in the refugee and host populations.

The Limitations section now clearly states that the findings may not be generalizable to all of Cox's Bazar or to populations using other healthcare providers.

We have also removed overly broad claims regarding global outbreak magnitude.

**Change made:**

Revised Study Setting and Population, Discussion, and Limitations.

**Major Comment 7. Inconsistency in age distribution**

**Comment:**

The age-group counts and percentages in Table 1 appeared inconsistent.

**Response:**

Thank you. We carefully rechecked the age-group counts and percentages and corrected the inconsistencies.

The revised values are:

- 0–14 years: 16,371 (46.0%)
- 15–29 years: 10,141 (28.5%)
- 30–59 years: 7,655 (21.5%)
- ≥60 years: 1,414 (4.0%)

**Change made:**

Table 1 and Table 2 corrected and cross-checked.

**Major Comment 8. Clinical severity underestimation**

**Comment:**

The low proportion of severe dengue and warning signs may reflect early detection or RDT-only inclusion.

**Response:**

We agree that the observed severity distribution should be interpreted within the context of facility-based, RDT-confirmed surveillance. The revised manuscript now emphasizes the potential for selection and surveillance bias resulting from exclusion of mild/asymptomatic infections that did not present for care and clinically suspected RDT-negative cases.

Admission is also no longer interpreted as a direct proxy for biological disease severity. It is explicitly described as a clinical and programme-management outcome influenced by both clinical and operational factors.

**Change made:**

Revised Methods, Discussion, and Limitations.

**Major Comment 9. No serotype/genotype data**

**Comment:**

The lack of serotype/genotype data limits interpretation of outbreak dynamics.

**Response:**

We agree. The revised manuscript explicitly states that molecular confirmation, serotyping, and genotyping were unavailable. We now note that this prevented assessment of whether circulating viral strains or serotypes contributed to temporal or clinical differences.

**Change made:**

Expanded Diagnostics and Limitations sections.

**Major Comment 10. Figure clarity**

**Comment:**

Figures lacked confidence intervals and explanation of model fits.

**Response:**

We have expanded the Figure 3 legend to clearly explain the observed weekly counts, phase-specific Poisson regression fits, and the vertical line defining the annual peak separating growth and decay phases. Model estimates and 95% confidence intervals are presented in Table 3.

**Change made:**

Expanded Figure 3 legend and linked interpretation to Table 3.

**Minor Comments**

**Comment:**

Grammar, possessive errors, long sentences, inconsistent terminology, and repetition.

**Response:**

The manuscript has been extensively edited for grammar, clarity, sentence structure, terminology, and consistency. The incorrect possessive construction has been corrected, several long sentences have been shortened, terminology has been standardized, and repetitive policy-oriented statements have been reduced.

**Reviewer 2**

We sincerely thank Reviewer 2 for the detailed overall assessment and constructive recommendations. The major issues raised by the reviewer—missing data, model specification, denominator calculations, surveillance bias, RDT limitations, table inconsistencies, overinterpretation of sustained transmission, figure legends, and generalizability—have all been addressed in the revised manuscript.

**Missing data and regression model transparency**

The revised manuscript now reports variable completeness before modelling. Blood pressure measurements were available for 18,864 of 35,581 patients and were strongly age-dependent. Because complete-case analysis would have introduced substantial age-dependent selection, blood pressure was excluded from the primary model.

A new S3 Table summarizes completeness and missingness of variables considered in the primary analysis.

The final admission model included all 35,581 observations and 2,863 admission events, corresponding to approximately 204 admission events per estimated model parameter.

Model discrimination, calibration, and multicollinearity are now reported. The model showed:

- AUC = 0.828
- 95% CI = 0.818–0.837
- Hosmer–Lemeshow χ² = 42.92, df = 8, p<0.001
- adjusted GVIF range = 1.006–1.041

The significant Hosmer–Lemeshow result is interpreted cautiously in the context of the very large sample size, and the calibration plot is provided as S2 Figure.

**Denominator calculations**

Year-specific catchment denominators have been added and the annual crude reported dengue rates recalculated using the corresponding annual population estimates.

**Surveillance and selection bias**

The revised manuscript now clearly states that the analysis represents healthcare-attending, RDT-confirmed dengue cases and not all infections in the community.

**RDT limitations**

The manuscript now explicitly discusses the absence of product-specific sensitivity/specificity information, QA information, and molecular confirmation, and acknowledges potential misclassification, particularly among IgM-only cases.

**Tables and figures**

Age-group inconsistencies were corrected, missingness reporting was added, and figure legends were expanded.

**Interpretation of transmission dynamics**

Claims regarding endemicity have been substantially moderated. The revised Conclusion now states that recurrent outbreaks suggest the possibility of increasingly sustained transmission but that the available data do not establish endemicity.

**Limitations**

The Limitations section has been substantially expanded to address retrospective surveillance, selection bias, diagnostic limitations, incomplete mortality ascertainment, unmeasured confounding, clustering, generalizability, and limitations of the temporal modelling approach.

**Reviewer 3**

**Major Comment 1. IgM-only positivity and case definition**

**Comment:**

IgM positivity alone does not necessarily indicate acute infection. The reviewer requested sensitivity/specificity information, QA clarification, and discussion of possible misclassification.

**Response:**

We agree. Product-specific sensitivity and specificity, manufacturer, lot number, and lot-level QA information were not retained in the retrospective analytic database, and molecular confirmatory testing was not available.

We have therefore explicitly acknowledged these limitations in the Diagnostics and Limitations sections. The manuscript now cautions that IgM-only positivity may reflect persistent antibodies beyond the acute phase and may result in misclassification.

**Change made:**

Expanded Diagnostics, Discussion, and Limitations.

**Major Comment 2. Attack-rate calculations**

**Comment:**

Clarify denominator derivation, annual population estimates, refugee population changes, and host-community denominator estimation.

**Response:**

We agree. We now provide year-specific catchment population estimates for 2021–2024, explain the sources used for refugee and host-community estimates, and calculate annual crude reported dengue rates using year-specific denominators.

The derivation of these estimates is provided in S1 Table.

We also acknowledge that the annual programmatic denominators may not fully capture intra-year population movement and that formal uncertainty intervals were unavailable.

**Change made:**

Revised Data Analysis, Table 1 footnote, S1 Table, and Limitations.

**Major Comment 3. Potential selection bias**

**Comment:**

The manuscript should explicitly state that findings represent healthcare-attending cases and may underestimate community transmission.

**Response:**

We agree. The Study Setting and Population section now states that the dataset represents facility-detected dengue cases rather than all infections occurring in the refugee and surrounding host populations.

The Limitations section also states that mild/asymptomatic cases, individuals not seeking care, and clinically suspected RDT-negative cases were not captured.

**Change made:**

Revised Study Setting and Population and Limitations.

**Major Comment 4. Admission outcome influenced by programme decisions**

**Comment:**

Admission may reflect social factors, distance, referral systems, and bed availability rather than disease severity.

**Response:**

We agree and have revised the manuscript accordingly. Inpatient admission is now explicitly treated as a clinical and programme-management outcome rather than a direct proxy for biological disease severity.

We further state that admission may have been influenced by living circumstances, distance from facilities, referral pathways, and available inpatient capacity.

**Change made:**

Revised Data Analysis, Discussion, and Limitations.

**Major Comment 5. Logistic regression model requires more detail**

**Comment:**

Please report number of observations, missing-data handling, events-per-variable ratio, variable selection strategy, AUC confidence interval, and Hosmer–Lemeshow p-value.

**Response:**

We agree and have now reported all of these items.

The final model included 35,581 observations and 2,863 admission events. All variables included in the primary model were complete. Blood pressure was excluded because of substantial and strongly age-dependent missingness.

Covariates were selected a priori based on clinical relevance, WHO criteria, and potential epidemiological confounding.

The model had approximately 204 admission events per estimated model parameter.

Model performance was:

- AUC 0.828
- 95% CI 0.818–0.837
- Hosmer–Lemeshow χ²=42.92, df=8, p<0.001
- adjusted GVIF 1.006–1.041

**Change made:**

Revised Data Analysis and Results; added S2 Figure and S3 Table.

A calibration plot is provided in S2 Figure.

**Major Comment 6. Warning-sign variable causes circularity**

**Comment:**

The association between WHO warning signs and admission is expected because warning signs are part of admission criteria.

**Response:**

We agree. We now explicitly state in the Discussion that this association should not be interpreted as an independent biological predictor of severe disease because WHO warning signs are themselves used in clinical admission decisions.

In addition, we conducted a sensitivity analysis excluding the WHO warning-sign variable. The direction and magnitude of the main associations with age, sex, delayed presentation, refugee status, and chronic co-morbidity remained broadly consistent.

The sensitivity model is presented in S2 Table.

**Change made:**

Revised Discussion and added S2 Table.

**Major Comment 7. Strong conclusions regarding endemicity**

**Comment:**

The statement that dengue was “taking endemic form” was not sufficiently supported.

**Response:**

We agree and have moderated the language throughout the manuscript.

The revised Conclusion now states:

“The recurrent outbreaks and dengue case detection across successive years suggest the possibility of increasingly sustained transmission, although the available data do not establish endemicity.”

We also explicitly acknowledge the absence of entomological, seroprevalence, meteorological, and serotype data.

**Change made:**

Revised Discussion, Conclusion, and Limitations.

**Major Comment 8. Refugee versus host-community comparison**

**Comment:**

The association may be confounded by healthcare access, facility distribution, referral pathways, and socioeconomic factors.

**Response:**

We agree. The revised Discussion now states that the higher adjusted odds of admission among refugee patients should be interpreted cautiously and that residual confounding from healthcare access, facility distribution, referral pathways, distance to inpatient services, living circumstances, and socioeconomic factors cannot be excluded.

**Change made:**

Expanded Discussion and Limitations.

**Major Comment 9. Diagnostic timing analysis is biologically expected**

**Comment:**

The relationship between delayed presentation and IgM positivity is expected and contributes limited novelty.

**Response:**

We agree that the observed pattern is biologically expected. We have therefore reframed this finding as an operationally relevant diagnostic pattern rather than a novel biological discovery.

The Discussion emphasizes its practical implication for diagnostic selection in humanitarian settings, while also acknowledging the limitations of IgM-only positivity and the absence of molecular confirmation.

**Change made:**

Revised interpretation in the Discussion.

**Major Comment 10. Missing spatial analysis**

**Comment:**

The reviewer recommended a camp-level incidence map, hotspot analysis, and yearly spatial distribution.

**Response:**

We appreciate this valuable suggestion. A formal camp-level spatial/hotspot analysis was not added in the present revision. The current revision focused on analyses that could be supported consistently across the finalized analytic dataset and validated population denominators.

We have therefore not made any spatial inference beyond the available geographic description and have strengthened the discussion of generalizability and catchment-based interpretation.

We agree that a dedicated camp-level spatial analysis would be valuable in future work.

**Reviewer 3 Minor Comments**

***Introduction: “its' largest displacement”***

**Response:** Corrected.

***Awkward sentence structure in Introduction***

**Response:** The relevant sentences were revised for clarity and readability.

***Atypical dengue presentation***

**Response:** The interpretation was revised and supported with additional references from Bangladesh and South/Southeast Asian settings.

***“excel spreadsheet”***

**Response:** Corrected to “Microsoft Excel spreadsheet.”

***Poisson model assumptions***

**Response:** The revised manuscript now clearly states that the models are descriptive and that overdispersion, temporal autocorrelation, and meteorological seasonality were not explicitly modelled. This limitation is also explicitly stated in the Limitations section.

***Epidemic onset definition***

**Response:** The revised Methods now defines the annual peak week and the corresponding growth and decay phases explicitly.

***Table 1 inconsistencies***

**Response:** The age-group counts and percentages were rechecked and corrected.

***“The median age of the cases were”***

**Response:** Corrected to “The median age of the cases was.”

***“8% (2,863 cases) necessitated inpatient admission”***

**Response:** Revised to clearer wording indicating that 8.0% (2,863/35,581) were admitted for inpatient care.

***Blood pressure missingness***

**Response:** The extent and age-dependent pattern of missing blood pressure data are now reported in detail. Blood pressure was excluded from the primary multivariable model to avoid selection bias from complete-case analysis. S3 Table summarizes the missingness.

***Table 6 warning signs***

**Response:** A footnote has been added clarifying that WHO warning signs were not mutually exclusive and that individual patients could have more than one warning sign.

***Low CFR attributed to IOM response***

**Response:** The causal interpretation has been removed. The revised Discussion presents multiple possible explanations and explicitly acknowledges incomplete mortality ascertainment.

**Additional Analyses Suggested by Reviewer 3**

1. **Year-specific admission models:** Separate year-specific admission models were not added. Instead, calendar year was incorporated directly as an a priori covariate in the primary multivariable model to account for temporal differences in admission practices and outbreak phase.
2. **Camp-level hotspot mapping:** A formal hotspot analysis was not added in this revision. We agree that this would be valuable for future spatial epidemiological work.
3. **Age-specific epidemic curves:** Age-specific epidemic curves were not added in the current revision. The age distribution is presented descriptively, and age is included in the multivariable admission model.
4. **Monthly diagnostic positivity trends:** Monthly NS1 and IgM positivity trends were not added. Instead, the manuscript reports positivity patterns in relation to delay from symptom onset to presentation, which directly reflects the timing of diagnostic marker detection.
5. **Sensitivity analysis excluding IgM-only cases:** A separate sensitivity analysis excluding IgM-only cases was not included in the final revision. Instead, we strengthened the description of diagnostic uncertainty and potential misclassification among IgM-only positive cases and explicitly acknowledged the absence of molecular confirmation and product-specific test-performance data as limitations.

We thank the Editor and Reviewers again for their thoughtful and constructive comments. We believe that the revisions have substantially improved the methodological transparency, statistical rigor, clarity of reporting, and interpretation of the manuscript. We hope that the revised manuscript is now suitable for consideration for publication in PLOS Neglected Tropical Diseases.

Sincerely,

**Charls Erik Halder**

Corresponding Author

On behalf of all authors

Manuscript ID: PNTD-D-26-00603
