# Supplementary material for: Epidemiological, clinical, and diagnostic characteristics of a large-scale upsurge of dengue in the rohingya refugee camps and host communities in Cox’s Bazar, Bangladesh, 2021 to 2024: A retrospective study
Source: PLoS Negl Trop Dis. 2026 Oct 23;20(10):e0014198. doi: 10.1371/journal.pntd.0014198 (PMC13719038; doi:10.1371/journal.pntd.0014198)
Supplement: Peer review history

# Peer review: Decision Letter 0

**DOI:** 10.1371/journal.pntd.0014198.r001

**Authors:** Michael Holbrook, Md. Kamrujjaman

22 Jun 2026

PNTD-D-26-00603

Epidemiological, Clinical, and Diagnostic Characteristics of a Large-Scale Upsurge of Dengue in the Rohingya Refugee Camps and Host Communities in Cox’s Bazar, Bangladesh, 2021 to 2024: A Retrospective Study

PLOS Neglected Tropical Diseases

Dear Dr. Halder,

Thank you for submitting your manuscript to PLOS Neglected Tropical Diseases. After careful consideration, we feel that it has merit but does not fully meet PLOS Neglected Tropical Diseases's publication criteria as it currently stands. Therefore, we invite you to submit a revised version of the manuscript that addresses the points raised during the review process.

Please submit your revised manuscript within by Aug 19 2026. If you will need more time than this to complete your revisions, please reply to this message or contact the journal office at plosntds@plos.org. When you're ready to submit your revision, log on to https://www.editorialmanager.com/pntd/ and select the 'Submissions Needing Revision' folder to locate your manuscript file.

Please include the following items when submitting your revised manuscript:

* A letter that responds to each point raised by the editor and reviewer(s). You should upload this letter as a separate file labeled 'Response to Reviewers'. This file does not need to include responses to any formatting updates and technical items listed in the 'Journal Requirements' section below.

* A marked-up copy of your manuscript that highlights changes made to the original version. You should upload this as a separate file labeled 'Revised Manuscript with Track Changes'.

* An unmarked version of your revised paper without tracked changes. You should upload this as a separate file labeled 'Manuscript'.

If you would like to make changes to your financial disclosure, competing interests statement, or data availability statement, please make these updates within the submission form at the time of resubmission. Guidelines for resubmitting your figure files are available below the reviewer comments at the end of this letter.

As the corresponding author, your ORCID iD is verified in the submission system and will appear in the published article. PLOS supports the use of ORCID, and we encourage all coauthors to register for an ORCID iD and use it as well. Please encourage your coauthors to verify their ORCID iD within the submission system before final acceptance, as unverified ORCID iDs will not appear in the published article. Only the individual author can complete the verification step; PLOS staff cannot verify ORCID iDs on behalf of authors.

We look forward to receiving your revised manuscript.

Kind regards,

Md. Kamrujjaman, Ph.D

Academic Editor

PLOS Neglected Tropical Diseases

Michael Holbrook

Section Editor

PLOS Neglected Tropical Diseases

Shaden Kamhawi

co-Editor-in-Chief

PLOS Neglected Tropical Diseases

orcid.org/0000-0003-4304-636XX

Paul Brindley

co-Editor-in-Chief

PLOS Neglected Tropical Diseases

orcid.org/0000-0003-1765-0002

**Additional Editor Comments (if provided):**

**Journal Requirements:**

If the reviewer comments include a recommendation to cite specific previously published works, please review and evaluate these publications to determine whether they are relevant and should be cited. There is no requirement to cite these works unless the editor has indicated otherwise.

1) Please provide an Author Summary. This should appear in your manuscript between the Abstract (if applicable) and the Introduction, and should be 150-200 words long. The aim should be to make your findings accessible to a wide audience that includes both scientists and non-scientists. Sample summaries can be found on our website under Submission Guidelines:

https://journals.plos.org/plosntds/s/submission-guidelines#loc-parts-of-a-submission

2) We notice that your supplementary Table (S1Table) are included in the manuscript file. Please remove them and upload them with the file type 'Supporting Information'. Please ensure that each Supporting Information file has a legend listed in the manuscript after the references list.

3) We have amended your Competing Interest statement to comply with journal style. We kindly ask that you double check the statement and let us know if anything is incorrect.

**Reviewers' Comments:**

Reviewer's Responses to Questions

**Key Review Criteria Required for Acceptance?**

As you describe the new analyses required for acceptance, please consider the following:

**Methods**

-Are the objectives of the study clearly articulated with a clear testable hypothesis stated?

-Is the study design appropriate to address the stated objectives?

-Is the population clearly described and appropriate for the hypothesis being tested?

-Is the sample size sufficient to ensure adequate power to address the hypothesis being tested?

-Were correct statistical analysis used to support conclusions?

-Are there concerns about ethical or regulatory requirements being met?

Reviewer #1: (No Response)

Reviewer #2: The study objectives were generally well articulated and aligned with the overall scope of the investigation, particularly regarding the epidemiological, clinical, and diagnostic characterization of dengue in the Rohingya refugee setting. The retrospective surveillance-based design was appropriate for describing outbreak trends and identifying operationally relevant associations within a large humanitarian dataset. The study population was clearly defined, and the inclusion of cases across multiple health facilities over four years represents a notable strength. The very large sample size was sufficient to support descriptive analyses and multivariable modelling with adequate statistical power. In addition, several appropriate statistical approaches were applied, including Poisson regression for epidemic phase modelling and multivariable logistic regression for admission predictors. However, important methodological clarifications remain necessary before the findings can be fully interpreted with confidence. The handling of missing data was insufficiently described, particularly for variables with substantial incomplete observations such as blood pressure measurements. The rationale for selection of covariates in regression models should be more explicitly justified, and the possibility of collinearity among clinically related predictors should be discussed in greater depth. Additional clarification is also needed regarding attack rate calculations, denominator estimation, and surveillance coverage across refugee and host populations. While ethical approval and anonymization procedures were adequately described, the manuscript would benefit from a broader discussion of surveillance bias, healthcare access differences, and limitations inherent to retrospective outbreak-response databases.

Reviewer #3: The study design and sample size are appropriate, but major revison is required before acceptance.

**********

**Results**

-Does the analysis presented match the analysis plan?

-Are the results clearly and completely presented?

-Are the figures (Tables, Images) of sufficient quality for clarity?

Reviewer #1: (No Response)

Reviewer #2: The analyses presented were generally consistent with the stated analytical plan and addressed the primary objectives of the study. The epidemiological trends, clinical characteristics, diagnostic patterns, and regression findings were organized logically and presented in a coherent manner. The large dataset and multi-year surveillance period strengthened the descriptive value of the results. Figures illustrating epidemic trajectories and phase-specific transmission dynamics contributed meaningfully to understanding temporal outbreak patterns, and the regression analyses provided potentially useful operational insights regarding admission risk factors and diagnostic timing patterns. However, several concerns regarding completeness and accuracy of reporting should be addressed. Internal inconsistencies were observed in Table 1, particularly within age-group frequencies and percentages, raising concerns regarding data presentation accuracy. The extent of missing data across variables was not clearly reported, and the sample sizes included in multivariable analyses were not fully specified. Some interpretations, particularly regarding endemic transition and sustained transmission, appeared stronger than the available evidence directly supports. In addition, diagnostic findings based solely on rapid diagnostic tests require more cautious interpretation given the absence of molecular confirmation and the known limitations of RDT sensitivity and specificity. The figures were generally clear and relevant, although several figure legends would benefit from additional methodological detail to improve interpretability and reproducibility.

Reviewer #3: The analysis well aligned with the study objectives.

**********

**Conclusions**

-Are the conclusions supported by the data presented?

-Are the limitations of analysis clearly described?

-Do the authors discuss how these data can be helpful to advance our understanding of the topic under study?

-Is public health relevance addressed?

Reviewer #1: (No Response)

Reviewer #2: The conclusions were generally aligned with the descriptive findings presented in the manuscript and appropriately emphasized the operational importance of dengue preparedness in humanitarian settings. The study contributes valuable evidence regarding dengue epidemiology, clinical presentation, and diagnostic patterns within a large refugee population, and the public health relevance of the findings was clearly articulated. The emphasis on strengthened surveillance, community-based referral systems, integrated diagnostics, and vector control is supported by the overall observations reported in the dataset. In addition, the manuscript successfully highlights the importance of sustained preparedness strategies in protracted displacement contexts, which represents a meaningful contribution to the neglected tropical disease literature. However, several conclusions currently extend beyond the direct strength of the evidence presented. Statements suggesting transition toward endemicity and sustained transmission should be interpreted more cautiously, as no entomological surveillance, serotyping, seroprevalence, or molecular confirmation data were included. The limitations section partially addressed the absence of genotyping but did not sufficiently discuss several important methodological constraints, including surveillance bias, retrospective data limitations, missing data, variability in healthcare access, and exclusive reliance on rapid diagnostic tests. Greater caution should therefore be exercised when interpreting population-level transmission dynamics and causal inferences. With more balanced interpretation and expanded acknowledgment of study limitations, the conclusions would be substantially strengthened.

Reviewer #3: The conclusions are generally supported by the data and the public health relevance is clear.

**********

**Editorial and Data Presentation Modifications?**

Use this section for editorial suggestions as well as relatively minor modifications of existing data that would enhance clarity. If the only modifications needed are minor and/or editorial, you may wish to recommend “Minor Revision” or “Accept”.

Reviewer #1: (No Response)

Reviewer #2: Several editorial and presentation-related revisions are recommended to improve clarity, consistency, and scientific rigor. First, all descriptive tables should be carefully rechecked for internal consistency, as discrepancies were identified in age-group frequencies and percentages in Table 1. Variable definitions and denominator calculations should be clarified more explicitly, particularly for attack rate estimations and subgroup analyses. A dedicated summary of missing data across variables should also be added, alongside clarification regarding handling of incomplete observations in regression analyses. Figure legends would benefit from additional methodological detail, especially for the epidemic phase modelling and Poisson regression analyses. The manuscript would also benefit from standardization of terminology, including consistent use of “outbreak,” “upsurge,” and “transmission.” Several sections of the Discussion contain repetitive policy-oriented language that could be condensed to improve readability and maintain analytical focus. Furthermore, causal or ecological interpretations regarding endemicity and transmission persistence should be moderated throughout the manuscript. Minor grammatical inconsistencies and sentence-structure issues are present across multiple sections, and professional language editing is recommended to improve readability and flow. Finally, the limitations section should be substantially expanded to address retrospective surveillance limitations, selection bias, diagnostic constraints, and changes in surveillance intensity over time.

Reviewer #3: (No Response)

**********

**Summary and General Comments**

Use this section to provide overall comments, discuss strengths/weaknesses of the study, novelty, significance, general execution and scholarship. You may also include additional comments for the author, including concerns about dual publication, research ethics, or publication ethics. If requesting major revision, please articulate the new experiments that are needed.

Reviewer #1: Comments for Manuscript Number PNTD-D-26-00603

Major comments

1. Selection bias & case definition (Major concern)

Only RDT-confirmed cases were included.

This excludes:

Clinically suspected dengue

False negatives (especially late NS1 or early IgM windows)

Problem issues: Attack rates, Severity estimates, Diagnostic pattern interpretation

Suggested revision:

Explicitly discuss sensitivity/specificity limitations of NS1/IgM

Provide:

Testing algorithm flow (who gets tested?)

Proportion of suspected vs tested vs confirmed cases

Consider sensitivity analysis if data available

2. Denominator & attack Rate validity

Attack rates are reported (e.g., 514.3 per 10,000 person-years) but:

Population denominator (691,857) is unclear

Refugee population is dynamic (migration, births, mobility)

Suggested revision:

Clarify: Source and stability of population denominators; whether yearly denominators were adjusted;

Add uncertainty

3. Poisson regression for epidemic growth

Weekly counts modeled using simple Poisson regression

No adjustment for:

Overdispersion

Temporal autocorrelation

Seasonality (rainfall, temperature)

Suggested revision:

Test for overdispersion -consider negative binomial

If possible consider: Time-series models (e.g., GAM, ARIMA)

Clarify how epidemic start/end defined (subjective?)

5. Extremely low case fatality rate (<0.1%)

Only 4 deaths out of 35,581 cases reported

This is unusually low for large dengue outbreaks

Suggested revision:

Explicitly discuss:

1. Mortality surveillance completeness

2. Community deaths vs facility deaths

3. Avoid over-attributing success to intervention model

6. Confounding in regression models

But missing key confounders:

1. Socioeconomic status

2. Distance to facility

3. Camp-level clustering

4. Time (year/epidemic phase)

Suggested revision:

Include:

Year as covariate

Facility-level clustering (random effects if possible)

Clarify variable selection strategy beyond “a priori”

8. Generalizability

Data from IOM-supported facilities only (36 sites)

Not representative of: Entire Cox’s Bazar and Non-IOM healthcare access

Suggested revision:

Clearly state limited external validity

Avoid broad claims like “largest outbreak globally” unless justified

9. Inconsistency in age Distribution

Table 1 appears inconsistent (e.g., % vs counts mismatch for age groups)

Needs verification and correction

10. Clinical severity underestimation

Severe dengue only 0.4%

Warning signs ~10%

Likely due to: Early detection bias or RDT-only inclusion

12. No serotype / genotype data

Acknowledged limitation but: Critical for interpreting outbreak dynamics

13. Figure clarity

Figures (pages 14–16) are informative but: Lack confidence intervals and overlaid model fits not clearly explained

Minor comments

Grammar issues:

“its’ largest displacement” is incorrect possessive

Redundancy in conclusion (repeated policy statements)

Overuse of long sentences (reduce for readability)

Ensure consistent terminology:

“upsurge”, “outbreak”, “epidemic”

This manuscript presents a valuable and large-scale analysis of dengue epidemiology in a highly vulnerable humanitarian setting. The dataset is impressive and addresses an important evidence gap. However, there are several critical methodological and interpretational issues that limit the robustness of the conclusions. In particular, concerns around selection bias due to RDT-confirmed case inclusion, unclear population denominators for attack rate estimation, lack of adjustment for overdispersion in Poisson models, and potential residual confounding in regression analyses need to be addressed. Additionally, the extremely low case fatality rate requires further clarification regarding surveillance completeness. The diagnostic analysis largely confirms expected biological patterns and should be reframed accordingly. I recommend major revision focusing on methodological transparency, cautious interpretation of findings, and strengthening of analytical approaches.

Reviewer #2: This manuscript addresses a highly important and underexplored topic by describing the epidemiological, clinical, and diagnostic characteristics of dengue in the Rohingya refugee camps and surrounding host communities in Cox’s Bazar, Bangladesh. The study is strengthened by its exceptionally large surveillance dataset, multi-year observation period, and operational relevance within a complex humanitarian setting. The integration of epidemiological trends, clinical features, diagnostic timing patterns, and admission predictors provides potentially valuable information for outbreak preparedness and health-system planning in displaced populations. The manuscript also addresses a significant gap in the neglected tropical disease literature, particularly regarding dengue epidemiology in refugee settings where evidence remains limited.

Despite these strengths, several substantial issues currently limit the scientific robustness and interpretability of the findings. Important methodological concerns include insufficient reporting and handling of missing data, unclear attack rate calculations, limited discussion of surveillance-related biases, and overinterpretation of transmission dynamics beyond the direct evidence available. The regression modelling strategy requires additional clarification, particularly regarding variable selection, model assumptions, and interpretation of highly correlated clinical predictors. In addition, the manuscript relies entirely on RDT-confirmed cases without molecular confirmation or serotyping, which should be more carefully acknowledged when interpreting diagnostic and epidemiological findings. Several descriptive tables also contain inconsistencies that require correction before the data can be fully evaluated with confidence.

Overall, the manuscript has substantial potential and addresses an important public health issue with clear policy relevance. However, significant methodological clarification, revision of interpretations, strengthening of the limitations section, and correction of data presentation issues are required before the manuscript can be considered suitable for publication in a high-standard journal. A major revision is therefore recommended.

Reviewer #3: This is an important dataset from an underrepresented humanitarian setting and will contribute valuable knowledge on dengue burden and prevention. However, several revisions are necessary before publication, as outlined in the reviewer comments. I also strongly suggest adding a camp-based hotspot map in the revised manuscript.

**********

PLOS authors have the option to publish the peer review history of their article (what does this mean?). If published, this will include your full peer review and any attached files.

If you choose “no”, your identity will remain anonymous but your review may still be made public.

**Do you want your identity to be public for this peer review?**  For information about this choice, including consent withdrawal, please see our Privacy Policy.

Reviewer #1: **Yes:**  MOHAMMAD ASHRAFUL AMIN

Reviewer #2: No

Reviewer #3: No

[NOTE: If reviewer comments were submitted as an attachment file, they will be attached to this email and accessible via the submission site. Please log into your account, locate the manuscript record, and check for the action link "View Attachments". If this link does not appear, there are no attachment files.]

**Figure resubmission:**

While revising your submission, we strongly recommend that you use PLOS’s NAAS tool (https://ngplosjournals.pagemajik.ai/artanalysis) to test your figure files. NAAS can convert your figure files to the TIFF file type and meet basic requirements (such as print size, resolution), or provide you with a report on issues that do not meet our requirements and that NAAS cannot fix.

After uploading your figures to PLOS’s NAAS tool - https://ngplosjournals.pagemajik.ai/artanalysis, NAAS will process the files provided and display the results in the "Uploaded Files" section of the page as the processing is complete. If the uploaded figures meet our requirements (or NAAS is able to fix the files to meet our requirements), the figure will be marked as "fixed" above. If NAAS is unable to fix the files, a red "failed" label will appear above. When NAAS has confirmed that the figure files meet our requirements, please download the file via the download option, and include these NAAS processed figure files when submitting your revised manuscript.

**Reproducibility:**

To enhance the reproducibility of your results, we recommend that authors of applicable studies deposit laboratory protocols in protocols.io, where a protocol can be assigned its own identifier (DOI) such that it can be cited independently in the future. Additionally, PLOS ONE offers an option to publish peer-reviewed clinical study protocols. Read more information on sharing protocols at https://plos.org/protocols?utm_medium=editorial-email&utm_source=authorletters&utm_campaign=protocols

Attachment Submitted filename: Comments for Manuscript Number PNTD-D-26-00603.docx

Attachment Submitted filename: Dengue Rohingya paper comments.docx

# Author comment: Author response to Decision Letter 1

**DOI:** 10.1371/journal.pntd.0014198.r002

21 Sep 2026

Attachment Submitted filename: Response_to_Reviewers_PNTD-D-26-00603.docx

# Peer review: Decision Letter 1

**DOI:** 10.1371/journal.pntd.0014198.r003

**Authors:** Michael Holbrook

11 Oct 2026

Dear Dr. Halder,

We are pleased to inform you that your manuscript 'Epidemiological, Clinical, and Diagnostic Characteristics of a Large-Scale Upsurge of Dengue in the Rohingya Refugee Camps and Host Communities in Cox’s Bazar, Bangladesh, 2021 to 2024: A Retrospective Study' has been provisionally accepted for publication in PLOS Neglected Tropical Diseases.

Before your manuscript can be formally accepted you will need to complete some formatting changes, which you will receive in a follow up email. A member of our team will be in touch with a set of requests.

Please note that your manuscript will not be scheduled for publication until you have made the required changes, so a swift response is appreciated.

IMPORTANT: The editorial review process is now complete. PLOS will only permit corrections to spelling, formatting or significant scientific errors from this point onwards. Requests for major changes, or any which affect the scientific understanding of your work, will cause delays to the publication date of your manuscript.

Should you, your institution's press office or the journal office choose to press release your paper, you will automatically be opted out of early publication. We ask that you notify us now if you or your institution is planning to press release the article. All press must be co-ordinated with PLOS.

Thank you again for supporting Open Access publishing; we are looking forward to publishing your work in PLOS Neglected Tropical Diseases.

Best regards,

Michael R Holbrook, PhD

Section Editor

PLOS Neglected Tropical Diseases

Michael Holbrook

Section Editor

PLOS Neglected Tropical Diseases

Shaden Kamhawi

co-Editor-in-Chief

PLOS Neglected Tropical Diseases

orcid.org/0000-0003-4304-636XX

Paul Brindley

co-Editor-in-Chief

PLOS Neglected Tropical Diseases

orcid.org/0000-0003-1765-0002

***********************************************************

Reviewer's Responses to Questions

**Key Review Criteria Required for Acceptance?**

As you describe the new analyses required for acceptance, please consider the following:

**Methods**

-Are the objectives of the study clearly articulated with a clear testable hypothesis stated?

-Is the study design appropriate to address the stated objectives?

-Is the population clearly described and appropriate for the hypothesis being tested?

-Is the sample size sufficient to ensure adequate power to address the hypothesis being tested?

-Were correct statistical analysis used to support conclusions?

-Are there concerns about ethical or regulatory requirements being met?

Reviewer #3: The study objectives and methods are clear and appropriate. The sample size and statistical analyses are adequate to support the main findings. The authors have addressed my previous methodological concerns. I have no further concerns, and no additional analyses are required for acceptance.

**********

**Results**

-Does the analysis presented match the analysis plan?

-Are the results clearly and completely presented?

-Are the figures (Tables, Images) of sufficient quality for clarity?

Reviewer #3: The results are clearly presented and consistent with the analysis described in the Methods. The tables and figures are clear and adequately present the main findings. I have no further concerns regarding the Results section.

**********

**Conclusions**

-Are the conclusions supported by the data presented?

-Are the limitations of analysis clearly described?

-Do the authors discuss how these data can be helpful to advance our understanding of the topic under study?

-Is public health relevance addressed?

Reviewer #3: The conclusions are supported by the findings, and the study limitations are clearly acknowledged. The public health relevance and importance of the findings for dengue preparedness in humanitarian settings are also well discussed.

**********

**Editorial and Data Presentation Modifications?**

Use this section for editorial suggestions as well as relatively minor modifications of existing data that would enhance clarity. If the only modifications needed are minor and/or editorial, you may wish to recommend “Minor Revision” or “Accept”.

Reviewer #3: The authors have adequately addressed my previous comments, and the revised manuscript is clear and well presented.

**********

**Summary and General Comments**

Use this section to provide overall comments, discuss strengths/weaknesses of the study, novelty, significance, general execution and scholarship. You may also include additional comments for the author, including concerns about dual publication, research ethics, or publication ethics. If requesting major revision, please articulate the new experiments that are needed.

Reviewer #3: Thank you to the authors for carefully addressing my previous comments. The manuscript has improved substantially. The case definition and diagnostic limitations, denominator calculation, selection bias, regression methods, and interpretation of the findings are now much clearer. The limitations of the Poisson analysis have also been appropriately acknowledged, and the authors have reasonably explained why some of the suggested additional analyses were not performed. Overall, my previous concerns have been adequately addressed. The revised manuscript provides a valuable contribution to the limited evidence on dengue epidemiology in the Rohingya refugee setting. I have no further comments, and the manuscript can be accepted in its current form.

**********

PLOS authors have the option to publish the peer review history of their article (what does this mean?). If published, this will include your full peer review and any attached files.

If you choose “no”, your identity will remain anonymous but your review may still be made public.

**Do you want your identity to be public for this peer review?**  For information about this choice, including consent withdrawal, please see our Privacy Policy.

Reviewer #3: No

# Editor report: Acceptance letter

**DOI:** 10.1371/journal.pntd.0014198.r004

**Authors:** Michael Holbrook

Dear Dr. Halder,

We are delighted to inform you that your manuscript, "Epidemiological, Clinical, and Diagnostic Characteristics of a Large-Scale Upsurge of Dengue in the Rohingya Refugee Camps and Host Communities in Cox’s Bazar, Bangladesh, 2021 to 2024: A Retrospective Study," has been formally accepted for publication in PLOS Neglected Tropical Diseases.

We have now passed your article onto the PLOS Production Department who will complete the rest of the publication process. All authors will receive a confirmation email upon publication.

The corresponding author will soon be receiving a typeset proof for review, to ensure errors have not been introduced during production. Please review the PDF proof of your manuscript carefully, as this is the last chance to correct any scientific or type-setting errors. Please note that major changes, or those which affect the scientific understanding of the work, will likely cause delays to the publication date of your manuscript. Note: Proofs for Front Matter articles (Editorial, Viewpoint, Symposium, Review, etc...) are generated on a different schedule and may not be made available as quickly.

Soon after your final files are uploaded, the early version of your manuscript will be published online unless you opted out of this process. The date of the early version will be your article's publication date. The final article will be published to the same URL, and all versions of the paper will be accessible to readers.

For Research Articles, you will receive an invoice from PLOS for your publication fee after your manuscript has reached the completed accept phase. If you receive an email requesting payment before acceptance or for any other service, this may be a phishing scheme. Learn how to identify phishing emails and protect your accounts at https://explore.plos.org/phishing.

Thank you again for supporting open-access publishing; we are looking forward to publishing your work in PLOS Neglected Tropical Diseases.

Best regards,

Shaden Kamhawi

co-Editor-in-Chief

PLOS Neglected Tropical Diseases

Paul Brindley

co-Editor-in-Chief

PLOS Neglected Tropical Diseases
